# All muscarinic acetylcholine receptors (M_1_-M_5_) are expressed in murine brain microvascular endothelium

**DOI:** 10.1038/s41598-017-05384-z

**Published:** 2017-07-11

**Authors:** Beatrice Mihaela Radu, Antonio Marco Maria Osculati, Eda Suku, Adela Banciu, Grygoriy Tsenov, Flavia Merigo, Marzia Di Chio, Daniel Dumitru Banciu, Cristina Tognoli, Petr Kacer, Alejandro Giorgetti, Mihai Radu, Giuseppe Bertini, Paolo Francesco Fabene

**Affiliations:** 1 Department of Neuroscience, Biomedicine and Movement Sciences, University of Verona, Verona, 37134 Italy; 2 Department of Anatomy, Animal Physiology and Biophysics, Faculty of Biology, University of Bucharest, Bucharest, 050095 Romania; 3 Department of Public Health, Experimental and Forensic Medicine, University of Pavia, Pavia, 27100 Italy; 4 Department of Biotechnology, University of Verona, Verona, 37134 Italy; 5 Engineering Faculty, Constantin Brancusi‘ University, Calea Eroilor 30, Targu Jiu, 210135 Romania; 6 Department of Public Health and Community Medicine, University of Verona, Verona, 37134 Italy; 7 National Institute of Mental Health, Klecany, 25067 Czech Republic; 8 Department of Life and Environmental Physics, Horia Hulubei National Institute for Physics and Nuclear Engineering, PO Box MG-6, Reactorului 30, Magurele, 077125 Romania

## Abstract

Clinical and experimental studies indicate that muscarinic acetylcholine receptors are potential pharmacological targets for the treatment of neurological diseases. Although these receptors have been described in human, bovine and rat cerebral microvascular tissue, a subtype functional characterization in mouse brain endothelium is lacking. Here, we show that all muscarinic acetylcholine receptors (M_1_-M_5_) are expressed in mouse brain microvascular endothelial cells. The mRNA expression of M_2_, M_3_, and M_5_ correlates with their respective protein abundance, but a mismatch exists for M_1_ and M_4_ mRNA versus protein levels. Acetylcholine activates calcium transients in brain endothelium via muscarinic, but not nicotinic, receptors. Moreover, although M_1_ and M_3_ are the most abundant receptors, only a small fraction of M_1_ is present in the plasma membrane and functions in ACh-induced Ca^2+^ signaling. Bioinformatic analyses performed on eukaryotic muscarinic receptors demonstrate a high degree of conservation of the orthosteric binding site and a great variability of the allosteric site. In line with previous studies, this result indicates muscarinic acetylcholine receptors as potential pharmacological targets in future translational studies. We argue that research on drug development should especially focus on the allosteric binding sites of the M_1_ and M_3_ receptors.

## Introduction

While it is widely known that acetylcholine (ACh) is released by axonal terminals of cholinergic neurons, cholinergic receptors are not found solely on target neurons. Rather, these receptor subtypes are also expressed by multiple non-neuronal cell types, both in the CNS (central nervous system) and peripherally, including endothelial, epithelial, mesothelial and immune cells^1^. This is not surprising, since cholinergic regulatory mechanisms have been described even in organisms lacking nervous systems, including plants, algae, and bacteria^2^.

Of particular interest is the role of muscarinic cholinergic receptors (mAChR) in modulating brain circulation, as it is known that ACh plays an important regulatory role at the level of brain arterioles, capillaries and venules^3^. For example, anatomical evidence from rats shows that a significant portion of the projections from basal forebrain neurons to fronto-parietal cortical microvessels are cholinergic^4^. Additionally, it has been shown that intrinsic ACh neurons innervate intracortical cerebral blood vessels in rats^5^. More recently, it has been reported that the somatosensory cortex microvasculature is innervated by cholinergic neurons^6^. The functional effects, such as vasodilation, of ACh and equivalent agonists (e.g., carbachol) have been demonstrated on both the luminal and parenchymal sides of brain vasculature in humans and hamsters^7, 8^. However, the mechanisms by which ACh may act on the endothelium of brain microvasculature (independent of whether direct vascular innervation can be established), are not yet fully understood.

The exchange of signals between the blood stream and the brain parenchyma is facilitated by the close proximity of neurons or glial cells to the capillary anastomoses; in the mouse cortex, the average distance among the nearest blood capillary and neurons is 15 microns^9^. ACh has been shown to regulate peripheral vascular homeostasis, especially via actions on the endothelium^10^, for instance by stimulating epoxyeicosatrienoic acids production^11^. Indeed, cholinergic mechanisms in the mouse endothelium have been described in a variety of fresh tissue preparations of the macro- and microcirculation^12–15^. ACh has also been detected and quantified in the bloodstream, where it may interact with the luminal side of the endothelium^16^. Notably, however, endothelial cells present a high degree of heterogeneity in the expression of surface markers, carrier proteins, and intracellular enzymes, across and within tissues^17^.

The localization and functional activity of muscarinic and/or nicotinic ACh receptors in brain microvascular endothelia have been described humans, rats, and bovines^18–22^, but not in mice. Given the potential importance of this receptor family as non-neuronal drug targets^23^, and the increasing use of mAChR *knockout* mice as models for common neurological disorders^24–26^, a detailed characterization of mAChRs expression and functionality in the mouse brain microvasculature is a high priority.

The mAChRs are a sub-class of the G-protein-coupled receptors (GPCRs) family, comprising 5 subtypes (M_1_-M_5_). M_1_, M_3_, and M_5_ are coupled with the G_q_ protein and, via phospholipase C signaling pathway, generate cytosolic calcium transients; M_2_ and M_4_, on the other hand, couple with the G_i_ protein inhibiting the adenylyl cyclase^25, 26^. While obtaining mAChRs subtype-selective ligands is a primary goal in drug development^25, 27^, previous attempts have failed due to the highly conserved structure of the orthosteric binding site across the muscarinic receptor family members^28, 29^. On the other hand, the allosteric binding site seems to hold promise as a specific pharmacological target^27^. Yet, despite considerable recent progress in crystallography and molecular modeling of mAChRs (as well as the successful crystallization of human M_1_, M_2_, M_4_, and rat M_3_ receptors^30, 31^), no 3D structure predictions based on homology modeling studies have been carried out for mouse muscarinic receptors.

Indeed, previous studies of mAChRs expression in different species reported rather contradictory results, possibly due to the different cell sources and/or techniques used. Initially, using a radioligand binding assay, the presence of M_1_ and M_3_ was demonstrated in membrane samples from human and bovine microvessels^32^. However, subsequent ultrastructural investigations in rat capillaries showed a lack of mAChRs at this level; the few samples in which mAChR expression was detected on the perivascular astroglia and have been attributed to the endfeet of cholinergic neurons innervating the capillary basement membrane^19, 20^. In contrast, mAChRs have been reported in human endothelial cells from brain microvessels and capillaries^18^. In particular, the M_2_ and M_5_ receptors (and occasionally M_1_) have been identified in cells cultivated from intracortical biopsies obtained from patients with temporal lobe epilepsy. In addition, M_3_ and M_2_ receptors have been found in post-mortem fetal cerebral cortex samples (10–18 weeks of gestation). However, the peculiarity of these samples limits the generalization of the results.

Here, we report direct evidence that all 5 mAChRs subtypes are expressed in mouse brain microvascular endothelial cells using a combination of quantitative analyses of mRNA and protein levels. The M_1_ and M_3_ subtypes, in particular, show functional and pharmacological properties consistent with previous reports in the same tissue types from other species. While we confirm the abundant presence of M_3_ in mouse brain endothelial cells, similar to findings in other species, we report for the first time the expression, but low functional relevance, of M_1_ in these cells. We analyzed the conservation of the orthosteric and allosteric binding site among receptor subtypes along all annotated (as from GPCRdb^33^) eukaryotic species. Our data confirm the idea that, while the orthosteric cavity is highly conserved, the allosteric site is highly variable and thus represents a potential pharmacological target for drug design. Our findings support further translational and experimental cerebrovascular studies in mice.

## Results

### All muscarinic acetylcholine receptor subtypes are expressed by mouse brain microvascular endothelial cells

**Figure 1:**
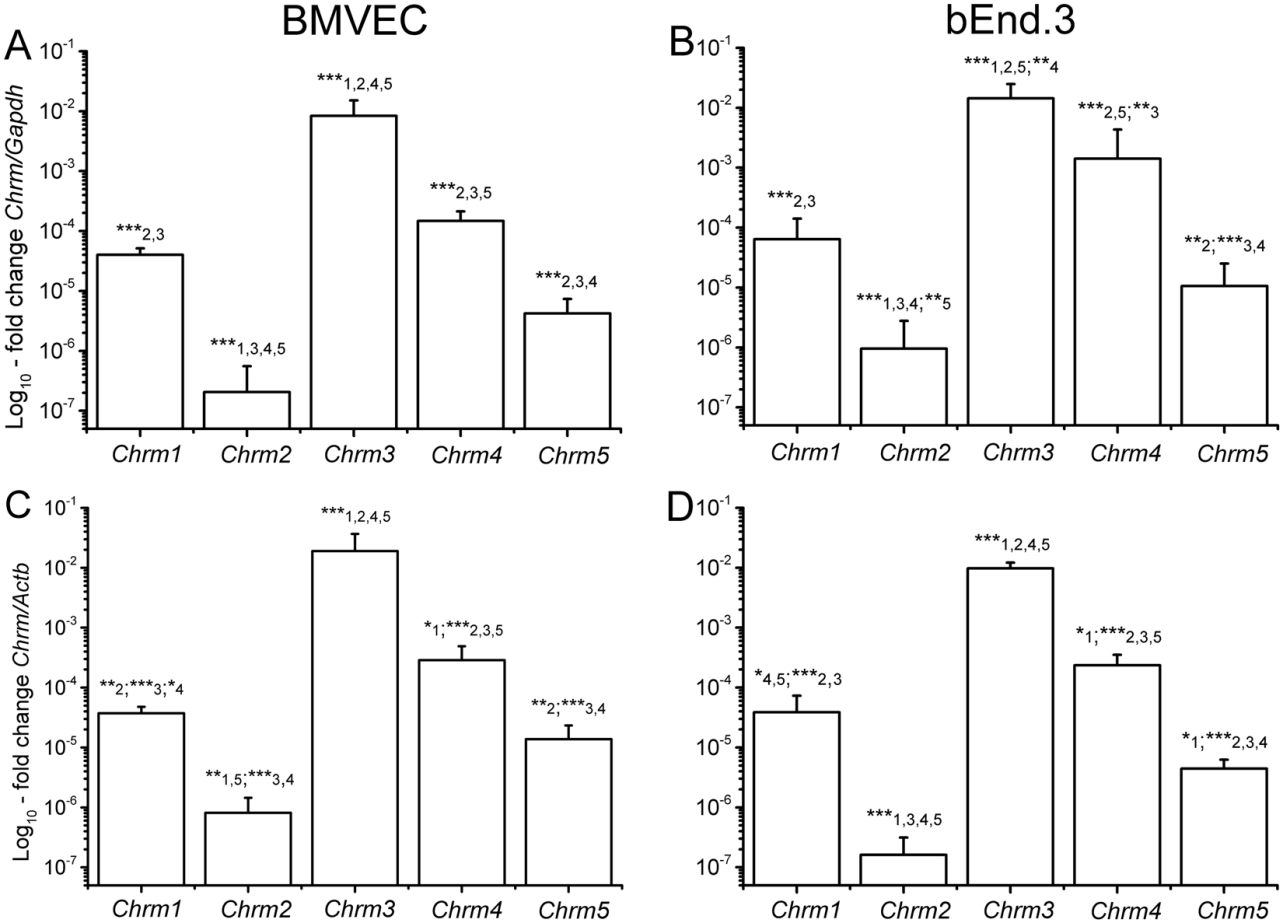
mRNA levels for *Chrm1 - Chrm5*, respectively encoding the M_1_-M_5_ receptors, quantified via qRT-PCR in BMVECs (**A,C**) and bEnd.3 cells (**B,D**). Data are normalized against two different reference genes, *Gapdh* (**A**,**B**) and *Actb* (**C**,**D**), and are plotted as mean ± SD (N = 5), from three different cell batches. One-way ANOVA analysis indicated: (**A**) F(32) = 80, *p* < 0.001; (**B**) F(25) = 56, *p* < 0.001; (**C**) F(25) = 58, *p* < 0.001; (**D**) F(14) = 121, *p* < 0.001. Statistical pairwise significance is indicated with asterisks (*0.01 < *p* < 0.05; **0.001 < *p* < 0.01; ***p < 0.001) followed by the compared variant.

We first sought to identify and quantify mRNA expression of mAChRs M_1_-M_5_ in two types of mouse brain microvascular endothelial cells. Quantitative real-time PCR (qRT-PCR) analyses using both β-actin (*Actb*) and glyceraldehyde 3-phosphate dehydrogenase (*Gapdh*) as reference genes indicated that both brain microvascular endothelial cells (BMVECs) and brain endothelial cells (bEnd.3) express mRNA transcripts for *Chrm1–5*, which encode mAChRs M_1_-M_5_. The ranking of mRNA levels in BMVECs and bEnd.3 cells was as follows: *Chrm3* > *Chrm4* $\tilde{ > }$ *Chrm1* $\tilde{ > }$ *Chrm5* > *Chrm2* (Fig. 1A–D). These rankings were consistently observed when data were normalized against either reference gene, despite the different functional roles of the latter. The most abundant muscarinic receptor gene, *Chrm3*, was approximately 10^5^ times more abundant than *Chrm2*, and about 200 times, 500 times and 3000 times more than *Chrm4, Chrm1* and *Chrm5*, respectively.

**Figure 2:**
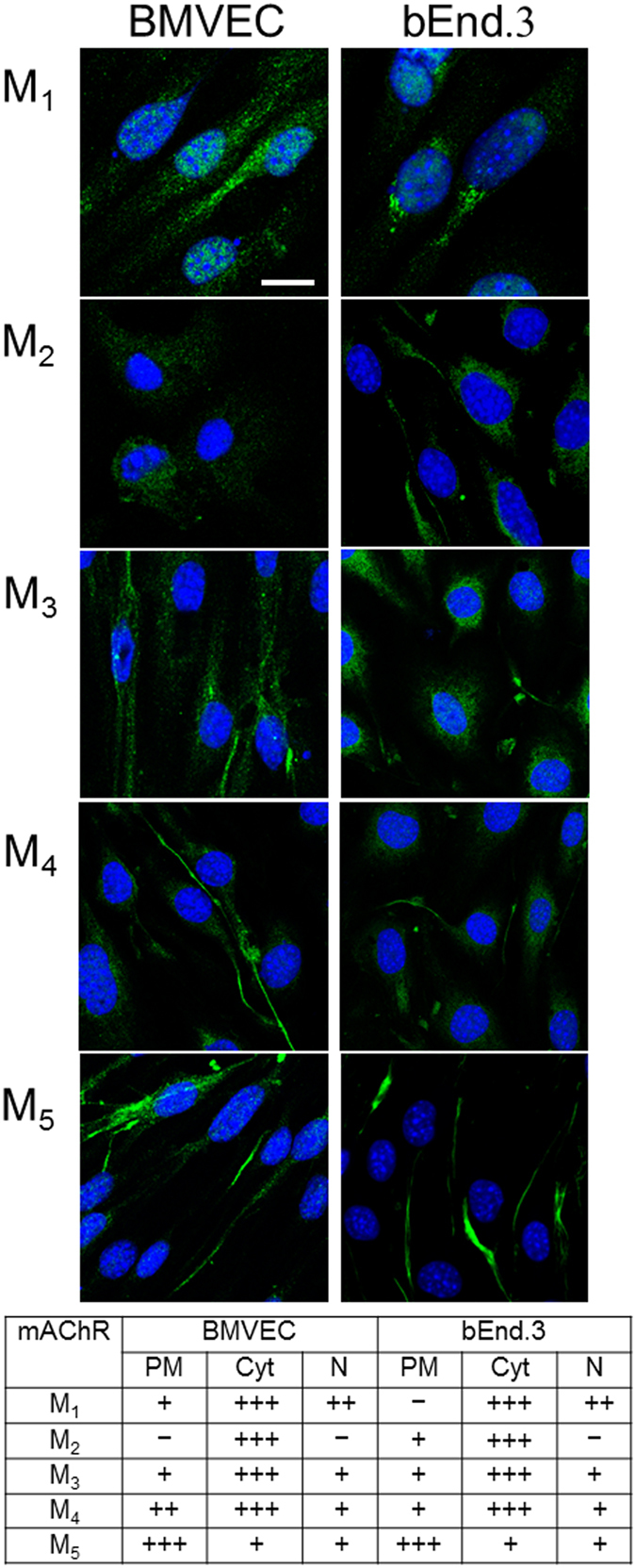
Detection of M_1_-M_5_ receptors by means of immunofluorescence in BMVECs and bEnd.3 cells. The labeling obtained with anti-M_1–5_ antibodies (green) is here merged with TO-PRO^®^-3 nuclear staining (blue); scale bar: 10 µm. The table shows qualitative estimates of protein abundance in the plasma membrane (PM), cytoplasm (Cyt), and nucleus (N). The evaluation was based on visual assessment of green fluorescence intensity on 40 cells per antibody, scored from not observable (−) to very intense (+++).

We next confirmed the presence of all muscarinic receptor subtypes and their subcellular distribution in both BMVECs and bEnd.3 cells via immunofluorescence staining (Fig. 2). A qualitative analysis did not reveal substantial differences in receptor expression between the two cell types; however, each subtype showed a specific subcellular localization pattern (Table in Fig. 2). In particular, most subtypes (M_1_-M_4_) are strongly present in the cytoplasm (mainly in the perinuclear region), while M_5_ was much more concentrated in the plasmalemma. Interestingly, an important fraction of M_1_ was localized in the nucleus, and was almost absent from the plasma membrane. M_3_ was abundant in the cytosol, but its presence partly extended to the plasma membrane.

### M_1_, M_3_, and M_4_ are the most abundant receptors in brain microvascular endothelial cells

**Figure 3:**
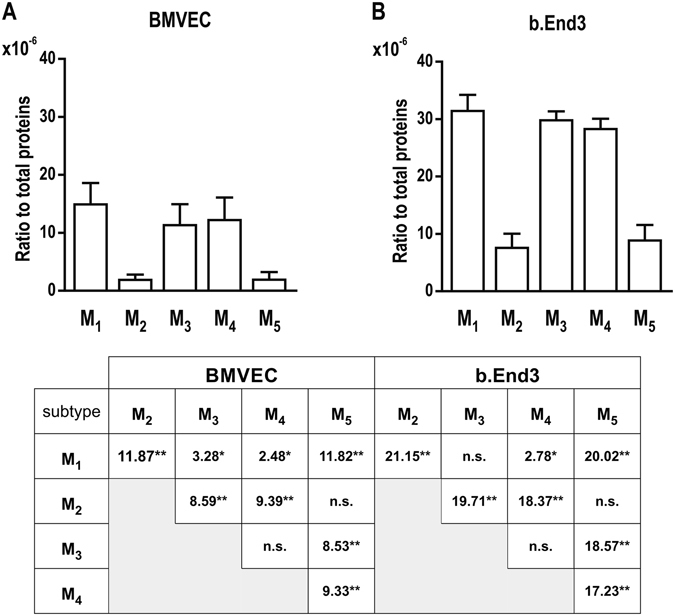
Relative protein abundance of M_1_-M_5_ receptors in brain microvascular endothelial cells (mean ± SD, N = 5). One-way ANOVA analyses showed significant differences between subtypes for both cell types: (**A**) F(64) = 62.03, *p* < 0.0001; (**B**) F(34) = 222.8, *p* < 0.0001. The Table shows the results (t values) of pairwise Holm-Sidak *post-hoc* tests (*p < 0.05; **p < 0.0001).

With the transcriptomic expression and subcellular distribution of mAChRs established, we next confirmed and quantified mAChR protein expression in mouse brain microvascular endothelial cells (BMVECs and bEnd.3 cells). Using the matrix-assisted laser desorption ionization time-of-flight (MALDI-TOF) mass spectrometry, we examined the amount of all muscarinic receptor proteins in brain microvascular endothelial cells (Supplementary Fig. 2C–G). Ratios of each mAChR (x10^−6^) normalized to the total amount of protein were calculated. One-way ANOVA analyses showed significant differences between subtypes for BMVEC cells (F(64) = 62.03, *p* < 0.0001) and b.End3 cells (F(34) = 222.8, *p* < 0.0001). Followed Holm-Sidak *post-hoc* tests indicated much higher levels of M_1_, M_3_ and M_4_ compared to M_2_ and M_5_ subtypes in both BMVECs (Fig. 3A) and b.End3 cells (Fig. 3B). Moreover, M_1_ expression was significantly higher than M_3_ and M_4_ in BMVECs significantly higher expression than M_4_ in bEnd.3 cells.

We also found that, for each mAChR subtype, expression in bEnd.3 cells was almost double that in BMVECs, and very similar results were obtained by means of the Bradford method (unpaired t-test, t = 8.384, df = 18, *p* < 0.0001, Supplementary Fig. 2B) thereby excluding potential biases due to overall differences in protein expression. Similar discrepancies in mAChR expression between primary and immortalized endothelial cells have been reported between freshly isolated and cultured bovine aortic endothelial cells^22^.

Protein quantitation data indicates that the M_1_ and M_4_subtypes are present in both cell types in amounts comparable to M_3_, in spite of significantly lower mRNA levels. A mismatch between mRNA and protein levels is not surprising *per se*, as such correlation in complex biological samples has been reported to be poor^34^. Different normalization factors between methods can play a role as well. Total protein loading control was reported to be more reliable than the housekeeping proteins loading controls^35^. We also examined the levels of β-actin and α-tubulin to see if they can be used as a reliable loading control under our experimental conditions. In the calibration procedure, we found that the protein expression level for β-actin is ~3.8 times higher in BMVEC than in bEnd.3 cells, the level of α-tubulin is ~5.6 times higher. Therefore, the total amount of protein in our samples was used as the normalization factor.

### Cholinergic activation of calcium signaling pathways in brain microvascular endothelial cells depends on muscarinic, but not on nicotinic receptors

The finding of muscarinic receptor transcripts and proteins, together with their subcellular localization in brain microvascular endothelial cells, prompted the characterization of their functional properties, which we pursued by means of calcium imaging recordings. As already mentioned, M_1_, M_3_ and M_5_ have been shown to trigger calcium transients in the cytosol in response to the binding of specific muscarinic agonists^25^.

The typical response to ACh for both BMVECs and bEnd.3 cells began with a sharp peak, followed by a smaller but sustained response (see Supplementary Figs 3–5 for representative examples), particularly after higher ACh concentrations (1 µM and 10 µM; Supplementary Fig. 3). This is consistent with the slow but sustained influx through store-operated calcium channels located in the plasma membrane that follows the peak in muscarinic-mediated vascular endothelial Ca^2+^ signaling^36^. Since ACh is an agonist for both nicotinic and muscarinic receptors, we examined the contribution of each receptor type to the overall ACh-induced Ca^2+^ signal. To accomplish this, we used a double-pulse protocol, starting with the application of the specific agonist (either nicotine or muscarine) followed by a 10-minute washout period, and subsequent application of ACh as a positive control (Supplementary Fig. 6). The 10-minute delay was confirmed to be sufficiently long in control experiments where ACh was applied both before and after the washout, triggering responses of similar amplitude in both trials (Supplementary Fig. 6A,B). Muscarine administration (100 µM) induced an intracellular calcium signal amplitude similar to the one triggered by 10 µM ACh (Supplementary Fig. 6C,D), while nicotine (300 µM) did not produce any response (Supplementary Fig. 6E,F). These results demonstrate that nicotinic receptors did not contribute to ACh-dependent calcium mobilization in our preparation and that the elicited signal depended entirely on GPCR muscarinic receptors.

**Figure 4:**
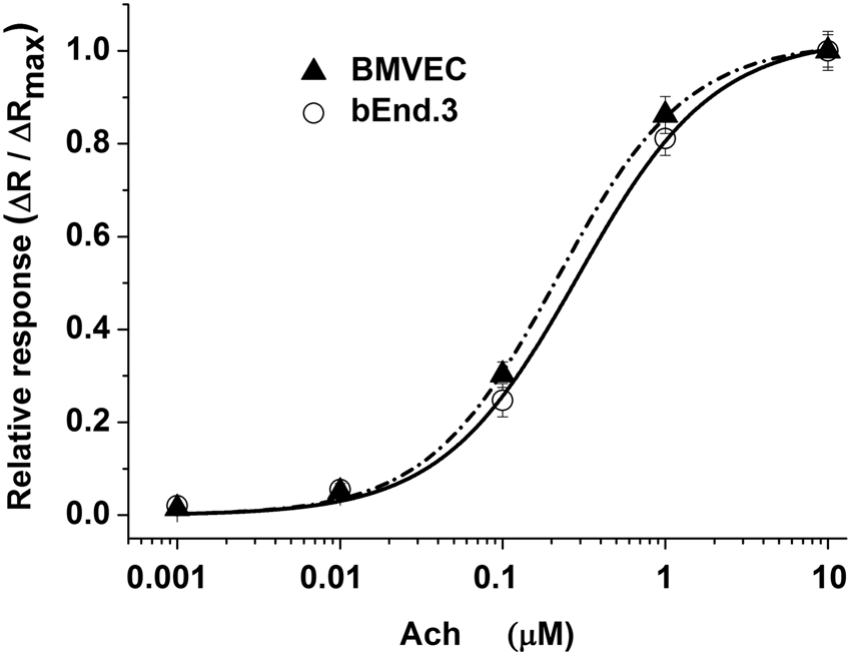
Concentration-response curves for ACh-induced increases in the normalized fluorescence ratio (ΔR/ΔR_max_) in BMVECs and bEnd.3 cells. Curves were fitted with eq. (1). Acetylcholine was used at concentrations ranging from 0.001–10 μM. Data are plotted as the mean ± SEM, N = 30 independent cells, from three different cell batches.

We further characterized muscarinic receptor functionality by investigating the concentration-response curve for ACh concentrations in the range of 1 nM to 10 µM. Increased ACh concentrations produced higher-amplitude calcium peaks (representative traces are shown in Supplementary Fig. 3). The best-fit curve for the data by the Hill equation (eq. (1), details in Methods section) was obtained with α = 1. The fit concentration-response curve yielded EC_50_ values of 0.216 ± 0.016 µM (mean ± S.E.M) for BMVECs and 0.290 ± 0.035 µM for bEnd.3 cells (Fig. 4).

Our data are in the same range as the EC_50_ values at 0.4 µM previously calculated for ACh-induced calcium signals in the HLE-B3 human lens epithelial cell line^37^. Interestingly, no direct correlation was observed between the abundance of M_1_-M_5_ receptor proteins (Fig. 3) and the EC_50_ curve left-shift (Fig. 4) or the maximal mean fluorescence ratio induced by ACh (Supplementary Fig. 3). Moreover, our results indicate that primary BMVEC cells are more sensitive to agonist exposure compared to the immortalized counterparts, their EC_50_ curve being left-shifted. These data are in accordance with the distinct Ach-induced calcium response of muscarinic receptors in primary cultivated vs. immortalized human lens epithelial cells^37^.

### Selective blocking of M_3_ receptors (but not M_1_) reduces ACh-induced calcium signals

**Figure 5:**
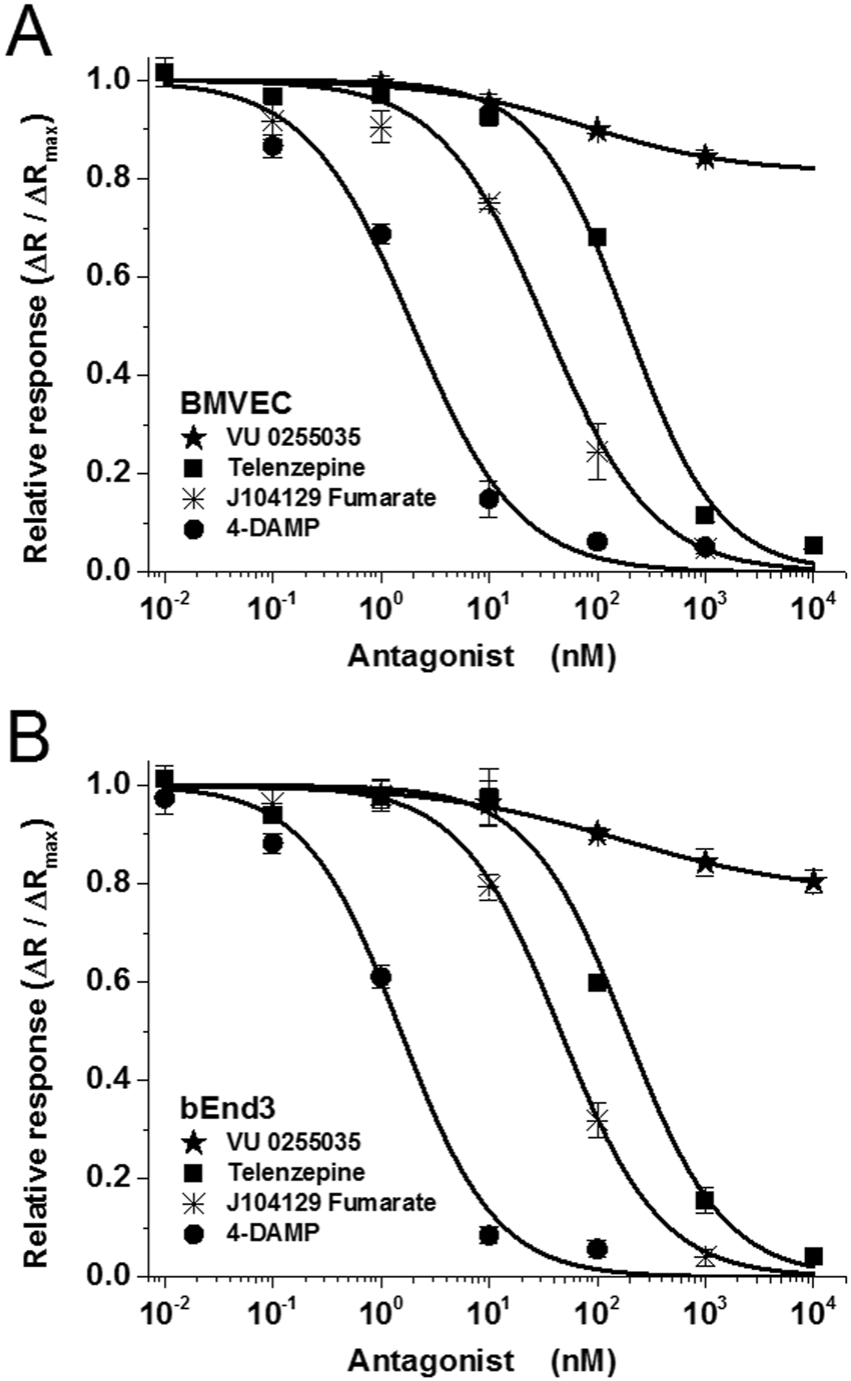
Inhibition curves of the response to acetylcholine (1 μM) in BMVECs (**A**) and bEnd.3 cells (**B**) in the presence of mAChR antagonists telenzepine, VU 0255035, J104129 fumarate, and 4-DAMP. Results are expressed as average fractions (±SEM on N = 40 independent cells) of the maximal fluorescence ratio (recorded at 10 µM ACh) produced by the lowest concentration in the tested range. Ratios (ΔR/ΔR_max_) are plotted as a function of antagonist concentration. Curves were fitted with eq. (2), and IC_50_ values are presented in Table 1.

The differential functional contribution of muscarinic receptor subtypes expressed by BMVECs and bEnd.3 cells was next investigated by calcium imaging, adding muscarinic orthosteric antagonists to the medium two minutes before ACh application. The results (Fig. 5) indicate that the M_1_ receptor antagonist VU 0255035 was ineffective in blocking ACh-induced calcium signals, while 3 other antagonists (M_3_ receptor antagonists J104129 fumarate and 4-DAMP, and M_1_ receptor antagonist telenzepine dihydrochloride hydrate) completely inhibited the signals triggered in response to 1 µM ACh (representative traces are presented in Supplementary Fig. 4 for bEnd.3 cells and in Supplementary Fig. 5 for BMVECs). The significant difference between the poor inhibition induced by the VU 0255035 and complete inhibition produced by telenzepine, both of them being accepted as M_1_ antagonists, can be explained by the difference in their affinity to M_3_ (8-fold less than for M_1_ in the case of telenzepine and 58-fold less for VU 0255035). Thus, at high concentrations, the former also inhibited M_3_ while the latter did not.

Inhibition curves for the 3 effective antagonists, when fit to a sigmoidal function (eq. (2)), yielded a Hill coefficient near 1 and absolute IC_50_ values in the range of 1 nM to 200 nM (Fig. 5 and Table 1). The maximal inhibition produced by VU 0255035 in the tested range of concentrations, as determined by the fit to eq. (2), was found to be less than 20%, and the relative IC_50_ was in the range of 75 nM to 150 nM. These data indicate that despite comparable protein expression levels for M_1_ and M_3_, the number of functional M_1_ receptors in the plasma membrane is much less compared to M_3_ receptors. Unfortunately, the lack of commercially available M_5_-specific antagonists limited our pharmacological characterization. The functional expression of mAChRs in BMVECs and bEnd.3 cells was also evaluated by applying agonists/antagonists targeting the allosteric site of the receptors. We found that the selective allosteric M_1_ antagonist MT-7 (20 nM)^38^ did not exert any inhibitory effect on the ACh-induced calcium signal in either type of brain microvascular endothelial cells (Supplementary Fig. 7A). In line with these findings, the selective allosteric M_1_ agonist^39^ AC-42 (10 µM), did not elicit any calcium signals (Supplementary Fig. 7B). Taken together, these results lead to the conclusion that only low levels of the functional form of the M_1_ receptors are present on the plasma membrane.

**Table 1:** Effects of 4 different muscarinic antagonists on the response of BMVECs and bEnd.3 cells to 1 μM ACh.

mAChR antagonist	BMVEC		bEnd.3		Published reports	
	IC_50_ (nM)	K_a_ (nM)	IC_50_ (nM)	K_a_ (nM)	Receptor	K_i_ (nM)
Telenzepine	191.29 ± 28.51	33.98	162.08 ± 21.86	36.43	M_1_ M_3_	2.5 20.7^74^
VU 0255035*	>10^4^	>1770	>10^4^	>2250	M_1_ M_3_	14.9 877**^52, 57^
J104129 fumarate	33.81 ± 3.80	6.00	43.31 ± 3.62	9.74	M_1_ M_3_	19 4.2^75^
4-DAMP	1.96 ± 0.48	0.42	1.39 ± 0.25	0.31	M_1_ M_3_	1 0.6^58, 76^

Absolute IC_50_ values and affinity constants K_a_ were calculated based on eqs (2) and (3), respectively, from the data presented in Fig. 5. For comparison, previously reported affinity constants for M_1_ and M_3_ receptors (K_i_), determined on individual clones expressed in testing cell lines, are also shown. *For VU 0255035, the absolute value of IC_50_ could not be established, due to this antagonist’s very low efficacy in blocking the calcium signal; **K_i_ values were determined via a radioligand binding assay^52, 57^, but the authors note that the K_i_ values obtained via a calcium mobilization assay could be at least two times higher than those determined in the radioligand binding assay.

### The variability of mouse muscarinic receptors lies in the allosteric binding site

We complemented our functional studies with a thorough conservation analysis of the residues located in the allosteric and orthosteric binding cavities. To such aim, we performed a multiple sequence alignment of all eukaryotic muscarinic receptors annotated in the GPCRdb database^40^.

**Figure 6:**
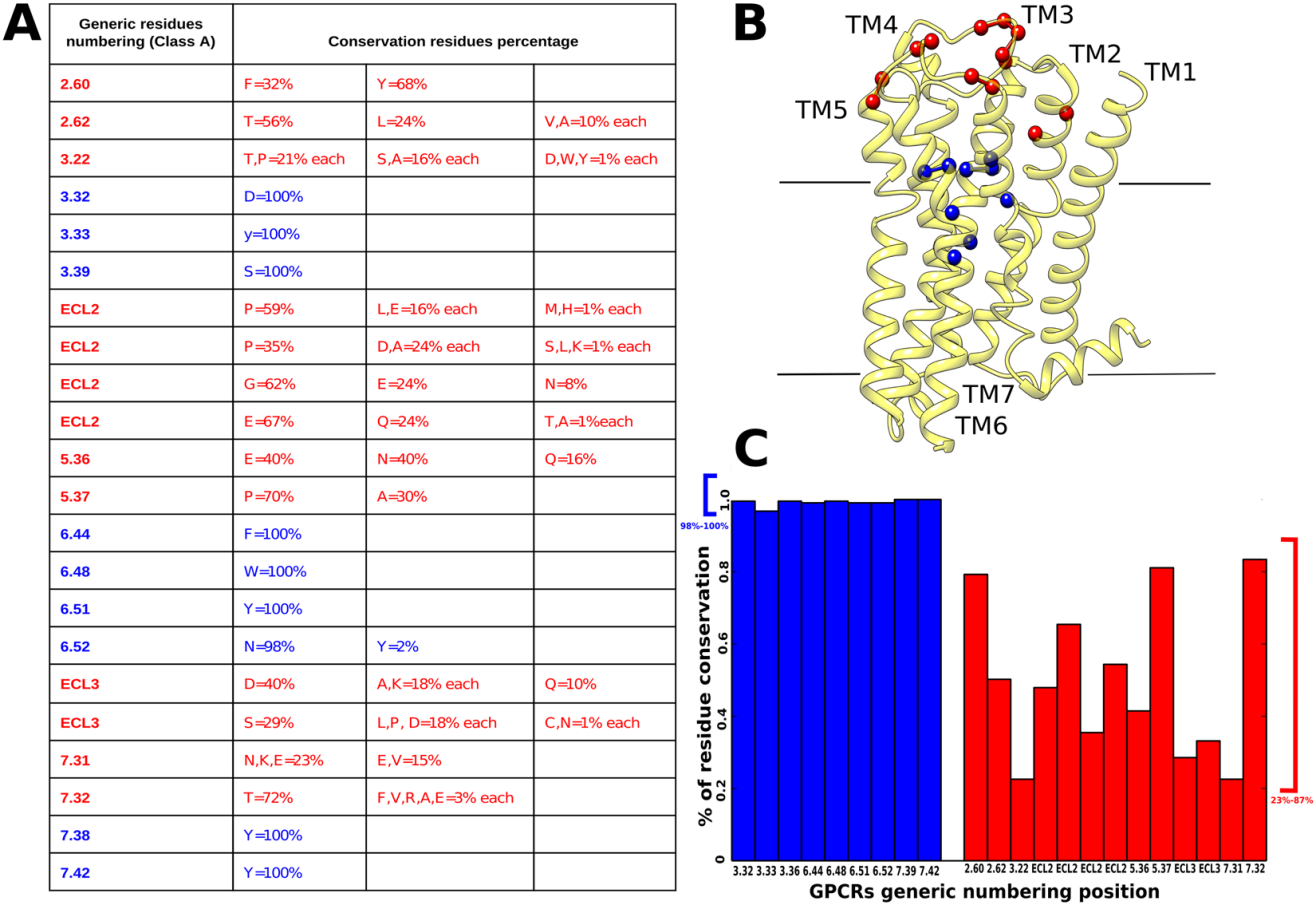
Conservation of orthosteric and allosteric site residues among annotated eukaryotic muscarinic receptors. (**A**) Conservation values of residues located in allosteric (red) and orthosteric (blue) binding sites. (**B**) Orthosteric and allosteric binding-site residues mapped on the M_3_ solved structure (PDB code: 4MQS). The residues are indicated as colored balls, following the same color code as in (**A**). (**C**) Plot of residues’ conservation values; residues of the orthosteric binding site (blue) and residues in the allosteric binding site (red).

To define orthosteric and allosteric residues, a “water level” was set in the intracellular half of the receptors as in Sandal *et al*.^41^ We validated this subdivision against the residues known to be involved in ligand-receptor interactions of both cavities in the crystal structures^42^. From our alignment, the orthosteric cavity reflects the full conservation of the residues^29, 43^ (Fig. 6A–C). Conversely, the residues present in the allosteric binding site^31^ emerge as highly variable (Fig. 6A–C). To the best of our knowledge, this is the first time that a conservation analysis has been performed considering muscarinic receptors along all annotated eukaryotic species. We then performed docking experiments on M_1_-M_5_ modeled mouse muscarinic receptors with the four antagonists (Supplementary Figures 8–11): Telenzepine, J104129 fumarate, VU 0255035 and 4-DAMP. In the case of M_5_ we docked also its highly specific antagonist: VU 0488130 (also called ML381^44^) (Supplementary Figure 12). Within the limitations of the method, it can be observed that, while three of these antagonists (Telenzepine, J104129 fumarate, 4-DAMP) interact with residues located mainly in the orthosteric binding site, VU 0255035 and VU 0488130, probably due to their larger size, interact also with residues positioned in the allosteric binding sites (Fig. 6, Supplementary Table 3). In particular, VU 0488130 may interact with residues located in the allosteric cavity that are exclusively present in the M_5_ receptor, i.e Q145 and K470. Hence, differences in affinities could be explained by the observation that the VU 0255035 and VU 0488130 networks of interactions involve less conserved regions (Supplementary Figures 11 and 12).

## Discussion

The results obtained in immortalized and primary cell cultures of the mouse brain microvasculature, including capillaries, lead to the conclusion that all muscarinic acetylcholine receptor subtypes are present in mouse brain endothelial cells. To our knowledge, this is the first report in which all mAChRs have been studied in terms of both mRNA expression and protein quantification in these cell types, thereby filling a gap in the existing literature, which focused on specific subsets. In addition, we provide a functional characterization of M_1_ and M_3_and argue that the distribution of M_1–5_ receptors may provide important insight into the endothelial response to cholinergic stimuli.

The analysis of transcripts revealed a gradient of mRNA expression by receptor subtype, with M_3_ representing the most abundant and M_2_ the lowest. In a previous study conducted in mouse retinal arterioles^14^, mRNA levels for the M_3_ receptor were similar to those reported here, but no other subtypes were detected. The discrepancy may be due to the substantial difference in detection thresholds, since in that study the cut-off was set at 10^−4^ relative to β-actin, which is above the levels we report here for the other 4 subtypes. Low levels of M_2_ (mRNA and protein abundance) in our brain microvascular endothelial cells are consistent with data reported recently for the proteome of mouse cerebral arteries^45^.

An important finding in the present work concerns the cellular localization of mAChRs as revealed by immunofluorescence. Aside from the plasma membrane, labeling was found in both the nucleus and the cytoplasm, with a subtype-specific distribution. This result fits within the framework of a novel paradigm that has emerged in the last ten years, indicating that GPCRs (the receptor family which mAChRs belong to) are able to engage in signaling even when positioned inside the cell, including the nucleus^46^. In particular, we found M_1_ mostly distributed in nuclear and cytoplasmic compartments, similarly to what has been reported for M_1_ in other cell types^47^, where the intracellular localization was explained either on the basis of constitutive internalization through a clathrin-dependent mechanism^48^ or via agonist exposure-dependent internalization^49^. In another report, the intracellular localization of M_1_ in neuroblastoma cells was considered associated with a distinct signaling pathway from the same receptors located in the plasma membrane^50^. On the other hand, the demonstration of intracellular mAChRs is not in itself proof of their involvement in specific alternative pathways. For one thing, receptor proteins must be present in the cytoplasm during their folding and maturation process until insertion in the plasmalemma. Additional experiments are necessary to describe the role of these intracellular fractions of mAChRs in brain endothelial cells.

Acetylcholine can trigger cytosolic calcium messenger pathways via both nicotinic and muscarinic receptors. The mechanisms of action of these receptors are very different; nicotinic receptor activation allows direct calcium ion influx from outside the cells, while the activation of Gq-coupled muscarinic receptors (M_1_, M_3_ and M_5_) triggers the inositol triphosphate calcium pathway. Our results show that nicotine could not elicit any calcium signaling in mouse brain microvascular preparations, which is in accordance with previous reports on rat coronary microvascular endothelial cells^51^. We therefore suggest that the measured calcium signals are due exclusively to the contribution of muscarinic acetylcholine receptors, e.g., M_1_, M_3_, and M_5_ receptors coupled to the phosphatidylinositol signaling pathway^1^, and not to nicotinic acetylcholine receptors. As M_2_ and M_4_do not contribute to the calcium signaling pathway, we did not include these receptors in the functional study. However, the high M_4_ protein expression levels, comparable to those of M_1_ (Fig. 3), prompt further investigations into the role of this receptor in brain endothelial cells function.

In spite of the very recent availability of an M_5_-specific antagonist, VU 0488130 (ML381)^44^, we opted to exclude M_5_ from our functional characterization, due to its very low expression levels compared to M_1_ or M_3_ (Figs 1 and 3). Moreover, an attempt to isolate the M_5_ response by saturating M_1_ and M_3_ with specific antagonists would be most likely unsuccessful because of the very small difference in the affinity of 4-DAMP to M_3_ and M_5_. In addition, no data are available on the affinity of J104129 fumarate to M_5_. According to Supplementary Table 1, the affinity of 4-DAMP to M_5_ is only twice as high as for M_3_which suggests that the antagonist, at concentrations saturating M_3_, will also strongly inhibit the M_5_ signal. We also report here that the selective blocking of M_1_ receptors does not reduce ACh-induced calcium signals. The functional expression of M_1_ receptors in brain microvascular endothelial cells is particularly important, not only because of their high relevance in murine models of neurological diseases^52–54^, but also because their role is often neglected in favor of their neuronal counterparts.

Our data show a mismatch between M_1_mRNA levels and protein abundance. Previous studies have described that the synthesis, trafficking and localization of muscarinic receptors is influenced by multiple mechanisms^40^, including chaperone-assisted synthesis and transport (for example, it has been shown that small G-protein ARF6 modulate trafficking of M_2_ receptor^40^). These processes might exert a major influence on how much mRNA is translated into functional proteins. In this context, one of the major findings revealed by the pharmacological characterization of mAChRs in our cells was the very low efficacy of VU 0255035 in inhibiting ACh-triggered signals. Considering the affinities of this antagonist for M_1_ and M_3_, we speculate that the small amount of inhibition we did observe was due to its binding to M_3_ and not to M_1_. This argument is supported by the fact that MT-7 (the most specific allosteric M_1_ antagonist, with no affinity for any mAChR subtypes other than M_1_^55^) also did not diminish the ACh signal. In turn, M_3_ antagonist J104129 fumarate completely inhibited the ACh signal over a range of concentrations corresponding to its specific affinity value. This strongly suggests that even if the protein expression of M_1_ was comparable to that of M_3_ (as shown by the MALDI-TOF data) the level of functional, plasma membrane-embedded M_1_ receptors is very low. This hypothesis is in accordance with previously reported values from mouse retinal arterioles^14^, where the presence of M_3_ was shown based on both mRNA quantification and a functional assay. In addition, the suggestion agrees with the low M_1_ levels revealed by immunofluorescence in the plasma membrane, with the majority of the protein being located near the nucleus. These data are further supported by the lack of M_1_ receptor activation in the presence of its very specific agonist, AC-42^56^.

Another key result of the present study is that the allosteric binding site of muscarinic receptors, including mouse receptors, is highly variable. Despite the commonly reported blocking specificity of J104129 fumarate, 4-DAMP, and telenzepine for M_3_ and M_1_, these antagonists can bind to all mAChRs, and the differences between their binding affinities for each receptor subtype are not very prominent^57, 58^ (Supplementary Table 1). From our molecular docking experiments, it can be appreciated that these three antagonists interact with highly conserved residues mainly located in the orthosteric binding site. We paid instead particular attention to VU 0255035 and VU 0488130 (a high affinity M_5_ antagonist) and found that they could both interact with residues located in both the orthosteric and allosteric sites. The variability in the allosteric site could explain the differences in antagonist binding affinities.

Early reports demonstrated the presence of M_1_, M_3_ and M_5_ in the human brain microcirculation^18, 32^, which makes a full characterization of all three subtypes in the mouse very important. We excluded M_5_ from our functional investigation because the data indicated low levels of both gene and protein expression, and there is limited knowledge on binding specificity of M_5_ antagonists^44^ to these receptors. The functional characterization that we performed for M_1_ and M_3_ receptors is physiologically relevant and in line with recent reports providing evidence on the importance of M_1_ and M_3_ in controlling blood pressure in various districts (i.e. mouse thoracic aorta and mesenteric artery^59^, rat mesenteric artery^60^, human umbilical vein^61^). Altogether, the present results provide an encouraging platform for future mouse-to-human translational studies. In particular, further experiments will be needed to fill a gap in our study, i.e. the functional characterization of the M_4_ subtype.

In summary, our findings highlight M_3_ receptors as major players in mouse brain microvasculature endothelium. On the other hand, the role of M_1_ receptors in microvasculature should not be discounted, in spite of its low functional contribution to the ACh-induced calcium signaling, since a high amount of protein was found expressed in the cells. As previously mentioned, the presence of this receptor family in brain microvasculature endothelia holds promise for the identification of novel non-neuronal drug targets. Our data specifically point out the M_1_ and M_3_ subtypes as interesting targets in translational studies when designing drugs that act on the blood brain barrier. Moreover, the levels of M_4_ protein expression found in our study, almost as high as M_1_, prompt further investigations that might extend our understanding of the role of muscarinic receptor subtypes in controlling brain endothelial cells.

## Methods

All experimental protocols were approved by the University of Verona ethical committee. The methods were carried out in accordance with the relevant guidelines and regulations.

### Brain endothelial cell cultures

To avoid biases due to contamination from astrocytes and/or terminals^18^, we studied mAChRs in primary cultures of endothelial cells separated from brain microvascular tissue or in an endothelial cell line. Balb/c mouse primary brain endothelial microvascular cells BMVECs (#PB-BALB-5023, PELOBiotech, Germany; passages 3–6) and cells from the Balb/c mouse cell line bEnd.3 (#ATCC® CRL-2299™, ATCC, USA; passages 20–30) were cultivated as previously described^62^. Both endothelial cell types were tested for mycoplasma contamination twice per year using the Hoechst (#B2883, Sigma) DNA staining protocol. Cells were plated at a density of 2 × 10^4^ cells/cm^2^ onto 24-mm cover glasses for the intracellular calcium imaging and immunofluorescence protocols, or into 25 cm^2^ flasks and further harvested for qRT-PCR and protein quantification assays.

### Gene expression via qRT-PCR

To quantify the expression levels of *Chrm1-5* (M_1_-M_5_ receptor-encoding genes) in mouse brain microvascular endothelial cells, total RNA was extracted using the GenElute Mammalian Total RNA MiniPrep Kit (RTN70, Sigma) according to manufacturer’s instructions. RNA concentrations were determined via spectrophotometric measurements of absorption at 260 and 280 nm (Beckman Coulter DU 730). DNase I treatment was used to remove contaminating genomic DNA. In agreement with manufacturer guidelines (Sigma), in our experiments the A_260_:A_280_ ratio was 2.03 ± 0.06 (bEnd.3) and 1.93 ± 0.03 (BMVEC). Reverse transcription was performed using the High-Capacity cDNA Archive Kit (Applied Biosystems, USA). *Actb* and *Gapdh* were used as housekeeping genes. The following mouse Taqman primers (Life Technologies, USA) were used in accordance with manufacturer guidelines: *Chrm1* (Mm00432509_s1), *Chrm2* (Mm01701855_s1), *Chrm3* (Mm01338409_m1), *Chrm4* (Mm00432514_s1), *Chrm5* (Mm01701883_s1), *Gapdh* (Mm999999_g1), and *Actb* (Mm00607939_s1). Further information on primer selection for *Chrm1* and *Chrm2* is provided in Supplementary Fig. 1. The relative abundance of *Chrm1-5* transcripts was assessed via qRT-PCR using the TaqMan methodology and the ABI Prism 7300 Sequence Detection System (Applied Biosystems, USA). Reactions were carried out in triplicate for 50 cycles.

### Assessment of muscarinic receptor localization via immunofluorescence and confocal microscopy

The cellular localization of the M_1_-M_5_ receptors was evaluated via immunofluorescence. Brain endothelial cells were treated with a blocking and permeabilization solution (0.3% Triton 100, 1% bovine serum albumin, 2% normal goat serum) for 30 min and were incubated overnight at room temperature with the primary antibody. They were then incubated for 1 h with the secondary antibody followed by an incubation with TO-PRO^®^-3 (1:3000, Life Technologies) for 10 min for nuclear staining, and were finally mounted with a fluorescent mounting medium. The following polyclonal rabbit primary antibodies from Alomone, Israel were used: anti-M_1_ (1:100, #AMR-001), anti-M_2_ (1:100, #AMR-002), anti-M_3_ (1:100, #AMR-006), anti-M_4_ (1:100, #AMR-004), and anti-M_5_ (1:100, #AMR-005). The primary antibody was omitted in negative-control samples. Goat anti-rabbit Alexa 488 (1:1000, Life Technologies) was used as the secondary antibody. Normal goat serum and fluorescent mounting medium were purchased from Dako, Milan, Italy.

Specimens were examined using a confocal fluorescence microscope (LSM 710, Carl Zeiss, Germany) equipped with a 63x oil objective. The following acquisition parameters were used: pinhole corresponding to 1 Airy Unit for each laser, 62 μm for the 488 nm laser and 66 μm for the 633 nm laser, digital gain of 1.00, and intensity of 5% for each laser. The acquisition parameter settings were kept fixed for each channel across different image acquisition sessions. Image acquisition was carried out using Zeiss LSM Image Browser software (Carl Zeiss, Germany). A visual evaluation of fluorescence intensity distribution in the nucleus, cytoplasm, and plasmalemma was used to characterize the cellular localization of the receptors. The green channel intensity was scored as follows: - (not observable), +(low), ++(medium) and +++(high). For each receptor were scored 40 cells. Because of the intrinsic difficulty in clearly isolating membrane labeling, we regard this evaluation as strictly qualitative.

### Protein extraction protocol

We employed a protein immunomagnetic affinity capture technique for the mass spectrometry-based quantification of M_1_-M_5_ receptors from BMVEC and bEnd.3 cell samples. Cells were harvested in microvials and immediately placed on ice. They were then homogenized with a series of 10 ultrasonic pulses (Sonopuls HD 2070, Bandelin, Germany), 1 sec each at 50% of the maximum frequency in 10 mM PBS (pH 7.4, Sigma) supplemented with the Protease Inhibitor Cocktail for use with mammalian cell and tissue extracts (#P8340, Sigma), according to the manufacturer’s instructions. Homogenates were subsequently centrifuged at 1000x*g* for 10 min at 4 °C to remove cellular debris. Cell lysates were collected, mixed with Laemmli loading buffer (#161-0737, Bio-Rad, Hercules, CA, USA) and heated at 70 °C for 20 min.

Using 4–15% Criterion™ TGX Stain-Free™ Protein Gels (#567-8084, Bio-Rad), we separated 15 μg of the lysate for each cell sample. The total protein profiles of the samples were then visualized using a Bio-Rad ChemiDoc MP imager within a few minutes after the completion of electrophoresis to check for protein sample and separation quality (Supplementary Fig. 2A). After visualization and image acquisition, UV activation of the bands was performed using the ChemiDoc MP system. Gels were split into individual lines, each containing separated proteins (from 10 to 250 kDa). Individual lines were then placed in vials containing 10 mL of distilled water. Next, the water solution and lines of gel were homogenized and consequently sonicated in an ultrasonic bath (Sonorex, Germany) for 20 min at 4 °C. Samples were then centrifuged at 6500xg (room temperature) and supernatants, filtered on a 0.2 mm PTFE microfilter were collected. A small volume of the supernatant was used to determine the total amount of proteins by the Bradford method (Supplementary Fig. 2B). Next, 1 mL of supernatant (i.e. 10% of the total) was incubated with antibody-coated magnetic particles that were prepared as described below.

We bound undiluted anti-M_1-5_ antibodies (1 µL, Alomone) to functionalized magnetic particles (Dynabeads® M-280, Tosyl-activated superparamagnetic polystyrene) for immunoaffinity capture. Protein standards for the M_1_-M_5_ receptors were provided by Alomone, Israel and were used as calibration controls in the matrix-assisted laser desorption ionization time-of-flight (MALDI-TOF) mass spectrometry analysis.

### Immunomagnetic isolation followed by MALDI-TOF MS analysis

We followed our previously published protocol^63^ with the antibodies detailed below. Candidate muscarinic receptors were isolated by using polyclonal affinity-purified antibodies purchased from Alomone Labs (as described above). The MALDI matrix 1,2-dimethoxy-4-hydroxycinnamic (sinapinic acid) and other chemicals were purchased from Sigma-Aldrich (St. Louis, MO, USA) and chemical solvents were purchased from Merck (Darmstadt, Germany).

The following buffers were used during the isolation procedure:

- Buffer A: 0.1 M sodium-phosphate buffer pH 7.4
- Buffer B: Phosphate-buffered saline (PBS) pH 7.4 with 0.1% (w/v) bovine serum albumin (BSA)
- Buffer C: 0.2 M Tris pH 8.5 with 0.1% (w/v) BSA;
- Buffer D: 100 mM glycine (pH 2.5)

Dynabeads M-280, tosyl-activated superparamagnetic polystyrene beads coated with polyurethane, were washed twice in Buffer A to remove sodium azide (NaN_3_) using magnetic particle concentrator following the manufacturer’s protocol (Thermo Fisher Scientific, Waltham, MA, USA). The above-listed antibodies (1 µL each) were dissolved in 100 µL of Buffer A and added to 100 µL suspension of Dynabeads, mixed for 1 min, followed by 24-hour incubation at 37 °C with mixing. Then, the supernatant was removed and the particles were washed twice with Buffer B (500 µL) at 4 °C. Free tosyl groups on the beads were blocked with Buffer C (500 µL; 4 h, 37 °C), followed by washing with Buffer B (500 µL; 5 min, 4 °C). Each individual preparation of the antibody-coated magnetic beads was resuspended in a 1 mL of supernatant and incubated with shaking for 1 h at 37 °C. The supernatants were then removed and the beads were washed five times with Buffer B (500 µL; 4 °C, 5 min, vortexing). The captured antigens were eluted with Buffer D (50 µL; 4 °C, 1 min, vortexing) and the beads were magnetically separated. The eluates were desalted using C18 ZipTip (EMD Millipore, Billerica, MA, USA) before analysis by matrix-assisted laser desorption ionization-time-of-flight mass spectrometry (MALDI-TOF MS).

### MALDI-TOF MS analysis

MALDI-TOF MS data were acquired on Autoflex mass spectrometer (Bruker Daltonics, Germany) with MALDI sample target (600 µm Chip™; Bruker Daltonics). Ionization was achieved by irradiation with a nitrogen laser (337 nm) operating at 4 Hz. Ions were accelerated at 20 kV with 250 ns of pulsed ion extraction delay. Freshly prepared 1,2-dimethoxy-4-hydroxycinnamic acid was used as matrix (10 mg/mL) in 50% acetonitrile 0.1% (v/v) of trifluoroacetic acid. The instrument’s parameters and laser energy were kept constant during a series of experiments performed on the same day for the comparison of intensity values (cps).

First, we obtained MS spectra of standard receptor proteins. The areas under the peak of interest were used to construct calibration curves, allowing later on the quantification of receptors in our samples. The protonated molecular ion peak (MH+) for each protein was detectable to a sub-pmol level with a signal-to-noise ratio >50. This detection limit was comparable with immunochemical assays.

The MS spectra obtained with our samples showed the MH+peaks at the expected m/z values, matching those observed with the standard proteins. The areas under the peak were then normalized against those interpolated from the calibration curves. The MALDI-TOF MS is a semiquantitative method; however, using rigorous sample preparation and data acquisition method, the intensity of the MH+peak(s) increased linearly with increasing quantities of each protein from nanomolar to picomolar ranges. Therefore, in this concentration range, the evaluations of protein biomarkers can be considered quantitative^63^.

### Intracellular calcium imaging measurements

Fura-2 AM-based Ca^2+^ imaging experiments were performed on brain microvascular endothelial cells as previously described^62^. Measurements were performed using a cooled CCD camera (Clara, Andor, Northern Ireland) and a Polychrome V monochromator (Till Photonics, Germany) coupled to a SliceScope (Scientifica, UK) upright fluorescence microscope with a 40x-water immersion objective (Olympus, Japan). Data acquisition was performed using Live Acquisition imaging software (Till Photonics, Germany), and calcium changes were determined based on the intensity ratio (R = I_340nm_/I_380nm_). Ratios were calculated for each cell in the microscope field and were based on individually defined regions of interest after background subtraction. Calcium imaging data were analyzed using Offline Analysis software (Till Photonics, Germany) and are plotted as the mean fluorescence ratio amplitude (ΔR = R − R_0_, where R_0_ represents the calcium signals recorded in unstimulated cells) ± SEM.

Solutions were delivered to brain endothelial cells through a 100 µm quartz perfusion head using an 8-channel valve pressurized system (ALA Scientific Instruments, USA). The Ringer’s solution contained (in mM) 140 NaCl, 5.6 KCl, 2 MgCl_2_, 2 CaCl_2_, 10 glucose, 10 HEPES (pH 7.4, adjusted with NaOH). Fura-2 acetoxymethyl ester (Fura-2 AM) and pluronic acid were purchased from Life Technologies, USA, and all common salts were from Sigma-Aldrich, Milan, Italy. Cytosolic Ca^2+^ transients resulting from the activation of muscarinic acetylcholine receptors were elicited by a 20-s pulse of 1 µM acetylcholine chloride (ACh, Sigma) application. The reproducibility of the effect of ACh was tested via a double-pulse protocol, with a 10-minute between-pulse Ringer’s washout period. In some experiments, we replaced the first pulse of ACh with a pulse of either (±)-muscarine chloride hydrate (#M104, Sigma) or (−)-nicotine hydrogen tartrate salt (#SML1236, Sigma) at previously described concentrations (100 µM and 300 µM, respectively)^51, 63^, followed by the second pulse (1 µM ACh) after a 10-min recovery period. Dose-response curves for the mAChR antagonists were obtained by preceding the second ACh pulse with a 2-min application of the antagonist. The following antagonists were used: J104129 fumarate (M_3_ antagonist, Tocris Bioscience, USA), VU 0255035 (selective M_1_ antagonist, Tocris Biosciences), 4-DAMP (1,1-dimethyl-4-diphenylacetyl piperidinium iodide; M_3_ antagonist, Sigma), and telenzepine dihydrochloride hydrate (M_1_ antagonist, Sigma). We also tested the effect of AC-42 (#SML0787, Sigma), a selective allosteric M_1_ agonist, with the EC_50_ = 29 nM (based on a Receptor Selection and Amplification Assays) for M_1_ and without any effect on M_2_-M_5_^56^. AC-42 was applied at its maximal activation concentration, based on the previously determined EC_50_ value of 0.2 µM evaluated by a calcium mobilization assay^39^. Moreover, we have tested the effect of the muscarinic toxin MT-7 (Smartox Biotechnology, France) at its saturation concentration, as previously described^55^, considering its high affinity for the M_1_ receptor, i.e. k_i_ = 0.2 nM for M_1_ and k_i_ > 2000 nM for M_2_-M_5_^55^.

### Homology modeling and molecular docking

The eucaryotic sequences were downloaded from the Pfam database (10.1093/nar/gkv1344), and the conservation of the residues were calculated as a percentage (X/P)*100, where X is the residue and P the length of the multiple sequence alignment. Since mouse M_1_-M_5_ lack structural information, homology modeling was carried out to predict their 3D structures. The sequences were retrieved from the Uniprot database, and the models were generated through the GOMoDo web-server^64^. To enforce the conserved structural fingerprints of class A (rhodopsin-like) GPCRs^65^, all sequence alignments between targets and templates (illustrated in detail in Supplementary Table 2) were verified manually. The 3D structures obtained in the homology modeling step were used to perform virtual docking studies using the HADDOCK docking program^66^ through the GOMoDo web-server. We used the GPCRdb generic number position^67^, which generalizes the classical Ballesteros-Weinstein numbering. Residues in positions 3.32, 3.33, 3.36, 3.37, 4.57, 5.39, 5.42, 6.48, 6.51, 7.39, and 7.43 were considered as ‘Active Residues’ to guide the docking. These residues have already been characterized as being involved in agonist/antagonist binding^33, 43^. The structures of the four antagonists were downloaded from the ZINC database^68^, and were parametrized with ACPYPE^69^. The docking protocol included three stages: (a) a rigid-body energy minimization, (b) a semi-flexible refinement in torsion angle space, and (c) a final refinement in an explicit solvent. We chose 1000 structures for the first step and 200 structures for the second and third docking steps, using default parameters. We selected the best structure for each docking as the one corresponding to the most populated cluster with the lowest energy value. Figures were generated with Chimera^70^.

### Data analysis

Quantitative RT-PCR data were obtained by normalizing *Chrm1*-*Chrm5* mRNA levels to those of *Gapdh* and *Actb* using the 2(-Delta C(T)) method, as previously described^71^. The results were expressed as log_10_-fold changes due to their extended scale distribution and were plotted as the mean ± SD.

The Hill formalism^37^ was used to fit both the ACh concentration–response and the inhibition curves determined via calcium imaging recordings from BMVECs and bEnd.3 cells. For the concentration-response curves, the following equation was used:

1 $${\rm{\Delta }}{\rm{R}}/{{\rm{\Delta }}{\rm{R}}}_{{\rm{\max }}}\,={C}^{{\rm{\alpha }}}/({{\rm{C}}}^{{\rm{\alpha }}}+{{\rm{EC}}}_{50}^{{\rm{\alpha }}})$$

C corresponds to the agonist concentration, ΔR_max_ is the amplitude ratio for the maximal response to 10 µM ACh, EC_50_ is the concentration of agonist required to elicit a half-maximal response, and α is the Hill coefficient describing cooperativity and mAChR receptor occupancy^37^. Inhibition curves resulting from the calcium imaging data analysis were fitted with the following equation:

2 $${\rm{\Delta }}{\rm{R}}/{{\rm{\Delta }}{\rm{R}}}_{{\rm{\max }}}=\,1+\,({\rm{X}}-1){{\rm{C}}}^{{\rm{\alpha }}}/({{\rm{C}}}^{{\rm{\alpha }}}+{{\rm{IC}}}_{50}^{{\rm{\alpha }}})$$

IC_50_ is the antagonist concentration required to reduce the agonist response by 50%, C is the agonist concentration (1 µM), α is the Hill coefficient. In equations (1) and (2) a Hill coefficient value of 1 indicates that only one molecule of agonist/antagonist is activating/inhibiting one receptor). X = 0 for telenzepine, J104129 fumarate and 4-DAMP, and X = the fitting parameter for VU 0255035.

The apparent dissociation constant was calculated using the Cheng–Prusoff equation, as follows:

3 $${{\rm{K}}}_{{\rm{a}}}={{\rm{IC}}}_{50}/(1+{\rm{C}}/{{\rm{EC}}}_{50})$$

Protein abundance was determined in the MALDI/TOF MS analysis, as described previously^72^. Briefly, the area under the curve of the most abundant mass band(peak) for each protein standard or sample was calculated in series of ten repetitions, and the computed average was used to determine the concentration of each muscarinic receptor protein. The mass band (peak) identification and quantification of each muscarinic receptor protein in our samples was determined in relation to the protein standard peak. Next, protein contents for each muscarinic receptor were normalized to the total amount of proteins in the sample.

Statistical analyses comparing the expression of the 5 receptor subtypes were carried out as follows. Differences in mRNA expression based on RT-PCR data were analyzed with one-way ANOVA (according to Yuan and colleagues^73^) followed by *post-hoc* Bonferroni tests. Protein quantification based on MALDI-TOF data were analyzed with one-way ANOVA followed by *post-hoc* Holm-Sidak 2 test. Analysis and data plotting were performed using OriginPro 8 (OriginLab Corporation, USA) and GraphPad Prism version 7.00 (GraphPad Software, La Jolla California USA).

### Data availability

The authors declare that all data supporting the findings of this study are available within the article and its Supplementary Information files or from the corresponding author upon reasonable request. The authors grant permission to Nature Publishing Group, a division of Macmillan Publishers Ltd, to publish all the images contained in the manuscript, under an Open Access license.

## Electronic supplementary material

Supplementary material

## References

[1] Eglen RM (2006). Muscarinic receptor subtypesin neuronal and non-neuronal cholinergic function. Auton. Autacoid Pharmacol..

[2] Wessler IK, Kirkpatrick CJ (2012). Activation of muscarinic receptors by non-neuronal acetylcholine. Handb. Exp. Pharmacol.

[3] Sato A, Sato Y, Uchida S (2004). Activation of the intracerebral cholinergic nerve fibers originating in the basal forebrain increases regional cerebral blood flow in the rat’s cortex and hippocampus. Neurosci. Lett..

[4] Vaucher E, Hamel E (1995). Cholinergic Basal Forebrain Neurons Project to Cortical Microvessels in the Rat - Electron-Microscopic Study with Anterogradely Transported Phaseolus-Vulgaris-Leukoagglutinin and Choline-Acetyltransferase Immunocytochemistry. J. Neurosci..

[5] Chedotal A, Cozzari C, Faure MP, Hartman BK, Hamel E (1994). Distinct Choline-Acetyltransferase (Chat) and Vasoactive Intestinal Polypeptide (Vip) Bipolar Neurons Project to Local Blood-Vessels in the Rat Cerebral-Cortex. Brain Res..

[6] Kuznetsova E, Schliebs R (2013). beta-Amyloid, cholinergic transmission, and cerebrovascular system–a developmental study in a mouse model of Alzheimer’s disease. Curr. Pharm. Des..

[7] Tsukahara T, Kassell NF, Hongo K, Vollmer DG, Ogawa H (1989). Muscarinic Cholinergic Receptors on the Endothelium of Human Cerebral-Arteries. J. Cereb. Blood Flow Metab..

[8] Rivers RJ, Duling BR (1992). Arteriolar Endothelial-Cell Barrier Separates 2 Populations of Muscarinic Receptors. Am. J. Physiol..

[9] Tsai PS (2009). Correlations of neuronal and microvascular densities in murine cortex revealed by direct counting and colocalization of nuclei and vessels. J. Neurosci..

[10] Cooke JP (2000). The endothelium: a new target for therapy. Vasc. Med..

[11] Campbell WB, Fleming I (2010). Epoxyeicosatrienoic acids and endothelium-dependent responses. Pflugers Arch..

[12] Peyter AC (2008). Muscarinic receptor M(1) and phosphodiesterase 1 are key determinants in pulmonary vascular dysfunction following perinatal hypoxia in mice. Am. J. Physiol. Lung Cell Mol. Physiol..

[13] Bagher P, Davis MJ, Segal SS (2011). Visualizing calcium responses to acetylcholine convection along endothelium of arteriolar networks in Cx40(BAC)-GCaMP2 transgenic mice. Am. J. Physiol. Heart Circ. Physiol..

[14] Gericke A (2011). Identification of the Muscarinic Acetylcholine Receptor Subtype Mediating Cholinergic Vasodilation in Murine Retinal Arterioles. Invest. Ophthalmol. Vis. Sci..

[15] Gericke A (2011). Role of M-1, M-3, and M-5 muscarinic acetylcholine receptors in cholinergic dilation of small arteries studied with gene-targeted mice. Am. J. Physiol. Heart Circ. Physiol..

[16] Kawashima K, Fujii T, Watanabe Y, Misawa H (1998). Acetylcholine synthesis and muscarinic receptor subtype mRNA expression in T-lymphocytes. Life Sci..

[17] Cines DB (1998). Endothelial cells in physiology and in the pathophysiology of vascular disorders. Blood.

[18] Elhusseiny A, Cohen Z, Olivier A, Stanimirovic DB, Hamel E (1999). Functional acetylcholine muscarinic receptor subtypes in human brain microcirculation: Identification and cellular localization. J. Cereb. Blood Flow Metab..

[19] Luiten PGM, deJong GI, VanderZee EA, vanDijken H (1996). Ultrastructural localization of cholinergic muscarinic receptors in rat brain cortical capillaries. Brain Res..

[20] Badaut J, Moro V, Seylaz J, Lasbennes F (1997). Distribution of muscarinic receptors on the endothelium of cortical vessels in the rat brain. Brain Res..

[21] Traish AM, Kim N, Carson MP, Detejada IS (1994). Characterization of Muscarinic Acetylcholine-Receptors in Cultured Bovine Aortic Endothelial-Cells. J. Recept. Res..

[22] Tracey WR, Peach MJ (1992). Differential Muscarinic Receptor Messenger-Rna Expression by Freshly Isolated and Cultured Bovine Aortic Endothelial-Cells. Circ. Res..

[23] Zhang YY (2014). Proteomics reveals potential non-neuronal cholinergic receptor-effectors in endothelial cells. Acta Pharmacol. Sin..

[24] Zhang, L. Cholinergic Receptor Knockout Mice in Animal Models of Cognitive Impairment (eds. Levin, E. & Buccafusco, J.) 199–222 (CRC Press/Taylor & Francis, Boca Raton, London, New York, 2006).

[25] Langmead CJ, Watson J, Reavill C (2008). Muscarinic acetylcholine receptors as CNS drug targets. Pharmacol. Ther..

[26] Wess J (2003). M-1-M-5 muscarinic receptor knockout mice as novel tools to study the physiological roles of the muscarinic cholinergic system. Recept. Channels.

[27] Conn PJ, Christopoulos A, Lindsley CW (2009). Allosteric modulators of GPCRs: a novel approach for the treatment of CNS disorders. Nat. Rev. Drug Discov..

[28] Felder CC, Bymaster FP, Ward J, DeLapp N (2000). Therapeutic opportunities for muscarinic receptors in the central nervous system. J. Med. Chem..

[29] Wess J (2005). Allosteric binding sites on muscarinic acetylcholine receptors. Mol. Pharmacol..

[30] Haga K (2012). Structure of the human M2 muscarinic acetylcholine receptor bound to an antagonist. Nature.

[31] Kruse AC (2012). Structure and dynamics of the M3 muscarinic acetylcholine receptor. . Nature.

[32] Linville, D. G. & Hamel, E. Pharmacological characterization of muscarinic acetylcholine binding sites in human and bovine cerebral microvessels. NaunynSchmiedebergs Arch. Pharmacol. **352**, 179–186 (1995). 10.1007/BF00176772 7477441

[33] Lu ZL, Hulme EC (1999). The functional topography of transmembrane domain 3 of the M1 muscarinic acetylcholine receptor, revealed by scanning mutagenesis. J. Biol. Chem..

[34] Maier T, Guell M, Serrano L (2009). Correlation of mRNA and protein in complex biological samples. Febs Lett.

[35] Colella AD (2012). Comparison of Stain-Free gels with traditional immunoblot loading control methodology. Anal. Biochem..

[36] Moccia F (2012). Store-Dependent Ca2 + Entry in Endothelial Progenitor Cells As a Perspective Tool to Enhance Cell-Based Therapy and Adverse Tumour Vascularization. Curr. Med. Chem..

[37] Collison DJ, Coleman RA, James RS, Carey J, Duncan G (2000). Characterization of muscarinic receptors in human lens cells by pharmacologic and molecular techniques. Invest. Ophthalmol. Vis. Sci..

[38] Fruchart-Gaillard C (2008). Different Interactions between MT7 Toxin and the Human Muscarinic M-1 Receptor in Its Free and N-Methylscopolamine-Occupied States. Mol. Pharmacol..

[39] Jacobson MA, Kreatsoulas C, Pascarella DM, O’Brien JA, Sur C (2010). The M1 Muscarinic Receptor Allosteric Agonists AC-42 and 1-[1 ′-(2-Methylbenzyl)-1,4 ′-bipiperidin-4-yl]-1,3-dihydro-2H-benzimidazol-2-one Bind to a Unique Site Distinct from the Acetylcholine Orthosteric Site. Mol. Pharmacol..

[40] Nathanson NM (2008). Synthesis, trafficking, and localization of muscarinic acetylcholine receptors. Pharmacol. Ther..

[41] Sandal M (2015). Evidence for a Transient Additional Ligand Binding Site in the TAS2R46 Bitter Taste Receptor. J. Chem. Theory. Comput..

[42] Kruse, A. C. *et al*. 4MQT Structure of active human M2 muscarinic acetylcholine receptor bound to the agonist iperoxo and allosteric modulator LY2119620. RCSB PDB, doi:10.2210/pdb4mqt/pdb (2013).

[43] Goodwin JA, Hulme EC, Langmead CJ, Tehan BG (2007). Roof and floor of the muscarinic binding pocket: Variations in the binding modes of orthosteric ligands. Mol. Pharmacol..

[44] Gentry PR (2014). Discovery, synthesis and characterization of a highly muscarinic acetylcholine receptor (mAChR)-selective M5-orthosteric antagonist, VU0488130 (ML381): a novel molecular probe. ChemMedChem..

[45] Badhwar A, Stanimirovic DB, Hamel E, Haqqani AS (2014). The proteome of mouse cerebral arteries. J. Cereb. Blood Flow Metab..

[46] Gobeil F (2006). G-protein-coupled receptors signalling at the cell nucleus: an emerging paradigm. Can. J PhysiolPharmacol.

[47] Lind GJ, Cavanagh HD (1993). Nuclear Muscarinic Acetylcholine-Receptors in Corneal Cells from Rabbit. Invest. Ophthalmol. Vis. Sci..

[48] Uwada J, Yoshiki H, Masuoka T, Nishio M, Muramatsu I (2014). Intracellular localization of the M1 muscarinic acetylcholine receptor through clathrin-dependent constitutive internalization is mediated by a C-terminal tryptophan-based motif. J. Cell Sci..

[49] Thomas RL, Langmead CJ, Wood MD, Challiss RAJ (2009). Contrasting Effects of Allosteric and Orthosteric Agonists on M-1 Muscarinic Acetylcholine Receptor Internalization and Down-regulation. J. Pharmacol. Exp. Ther..

[50] Uwada J, Anisuzzaman ASM, Nishimune A, Yoshiki H, Muramatsu I (2011). Intracellular distribution of functional M-1-muscarinic acetylcholine receptors in N1E-115 neuroblastoma cells. J. Neurochem.

[51] Moccia F, Frost C, Berra-Romani R, Tanzi F, Adams DJ (2004). Expression and function of neuronal nicotinic ACh receptors in rat microvascular endothelial cells. Am. J. Physiol. Heart Circ. Physiol..

[52] Sheffler DJ (2009). A Novel Selective Muscarinic Acetylcholine Receptor Subtype 1 Antagonist Reduces Seizures without Impairing Hippocampus-Dependent Learning. Mol. Pharmacol..

[53] Xiang ZX, Thompson AD, Jones CK, Lindsley CW, Conn PJ (2012). Roles of the M1 Muscarinic Acetylcholine Receptor Subtype in the Regulation of Basal Ganglia Function and Implications for the Treatment of Parkinson’s Disease. J. Pharmacol. Exp. Ther..

[54] Puri V, Wang XH, Vardigan JD, Kuduk SD, Uslaner JM (2015). The selective positive allosteric M1 muscarinic receptor modulator PQCA attenuates learning and memory deficits in the Tg2576 Alzheimer’s disease mouse model. Behav. Brain Res..

[55] Adem A, Karlsson E (1997). Muscarinic receptor subtype selective toxins. Life Sci..

[56] Spalding TA (2002). Discovery of an ectopic activation site on the M(1) muscarinic receptor. Mol. Pharmacol.

[57] Michel AD, Stefanich E, Whiting RL (1989). Direct Labeling of Rat M3-Muscarinic Receptors by [H-3] 4Damp. Eur. J. Pharmacol..

[58] Lazareno S, Buckley NJ, Roberts FF (1990). Characterization of Muscarinic M4 Binding-Sites in Rabbit Lung, Chicken Heart, and Ng108-15 Cells. Mol. Pharmacol..

[59] Yuan Q (2016). Maintenance of normal blood pressure is dependent on IP3R1-mediated regulation of eNOS. Proc. Natl. Acad. Sci. USA.

[60] Tangsucharit P (2016). Muscarinic acetylcholine receptor M1 and M3 subtypes mediate acetylcholine-induced endothelium-independent vasodilatation in rat mesenteric arteries. J. Pharmacol. Sci..

[61] Pujol Lereis VA (2006). Pharmacological characterization of muscarinic receptor subtypes mediating vasoconstriction of human umbilical vein. Br. J. Pharmacol.

[62] Radu BM (2015). Are they in or out? The elusive interaction between Qtracker (R) 800 vascular labels and brain endothelial cells. Nanomedicine (Lond).

[63] Hasuo H, Akasu T, Gallagher JP (1996). Muscarine activates a nonselective cation current through a M(3) muscarinic receptor subtype in rat dorsolateral septal nucleus neurons. J. Neurophysiol..

[64] Sandal, M. *et al*. GOMoDo: A GPCRs Online Modeling and Docking Webserver. Plos One **8**, (2013). 10.1371/journal.pone.0074092 PMC3772745 24058518

[65] Venkatakrishnan AJ (2013). Molecular signatures of G-protein-coupled receptors. Nature.

[66] De Vries SJ, van Dijk M, Bonvin AMJJ (2010). The HADDOCK web server for data-driven biomolecular docking. Nat. Protoc..

[67] Isberg V (2016). GPCRdb: an information system for G protein-coupled receptors. Nucleic Acids Res.

[68] Irwin JJ, Sterling T, Mysinger MM, Bolstad ES, Coleman RG (2012). ZINC: A Free Tool to Discover Chemistry for Biology. J. Chem. Inf. Model..

[69] Sousa da Silva, A.W. & Vranken, W.F. ACP. BMC Res. Notes **5**, 367 (2012). 10.1186/1756-0500-5-367 PMC3461484 22824207

[70] Pettersen EF (2004). UCSF chimera - A visualization system for exploratory research and analysis. J. Comput. Chem..

[71] Livak KJ, Schmittgen TD (2001). Analysis of relative gene expression data using real-time quantitative PCR and the 2(T)(-Delta Delta C) method. Methods.

[72] Schlosser G (2007). Coupling immunomagnetic separation on magnetic beads with matrix-assisted laser desorption ionization-time of flight mass spectrometry for detection of staphylococcal enterotoxin B. Appl. Environ. Microbiol..

[73] Yuan JS, Reed A, Chen F, Stewart CN (2006). Statistical analysis of real-time PCR data. BMC. Bioinformatics..

[74] Tanda G (2007). Effects of muscarinic M-1 receptor blockade on cocaine-induced elevations of brain dopamine levels and locomotor behavior in rats. J. Pharmacol. Exp. Ther..

[75] Mitsuya M (2000). Discovery of a muscarinic M-3 receptor antagonist with high selectivity for M-3 over M-2 receptors among 2-[(1S, 3S)-3-sulfonylaminocyclopentyl]phenylacetamide derivatives. Bioorg. Med. Chem..

[76] Dorje F (1991). Antagonist Binding Profiles of 5 Cloned Human Muscarinic Receptor Subtypes. J. Pharmacol. Exp. Ther..

