# Supplementary material for: All muscarinic acetylcholine receptors (M1-M5) are expressed in murine brain microvascular endothelium
Source: Sci Rep. 2017 Jul 11;7:5083. doi: 10.1038/s41598-017-05384-z (PMC5506046; doi:10.1038/s41598-017-05384-z)
Supplement: Supplementary file 1 — Supplementary material [file 41598_2017_5384_MOESM1_ESM.pdf]

Supplementary Material

for the paper

All muscarinic acetylcholine receptors (M_1-M_5) are expressed in murine brain microvascular endothelium

Beatrice Mihaela Radu, Antonio Marco Maria Osculati, Eda Suku, Adela Banciu, Grygoriy Tsenov, Flavia Merigo, Marzia Di Chio, Daniel Dumitru Banciu, Cristina Tognoli, Petr Kacer, Alejandro Giorgetti, Mihai Radu, Giuseppe Bertini, Paolo Francesco Fabene

Supplementary Table 1. Affinity constants of different antagonists for muscarinic acetylcholine obtained from the literature. The values for the affinity constants that match our experimental data for the effect of each antagonist on the total ACh-induced calcium signal recorded for BMVECs and bEnd.3 cells are shown in bold.

**Antagonist**	**Binding affinities for mAChR subtypes (nM)**					**M Receptor subtype**	**Biologic source**
	**M_1**	**M_2**	**M_3**	**M_4**	**M_5**		
Telenzepine	2.52	43.8	**20.7**	12.4	49.9	1	membranes from frozen rat brain[65]
						2-5	human receptors in CHO cells[65]
VU 0255035	14.87	661.33	876.93	1177.67	2362.33	1	CHO cells stably expressing rat receptor[46]
						2,3,5	CHO cells stably expressing human receptors[46]
						4	rat receptor expressed by stably transfected CHO-K1 cells[46]
J104129 fumarate	19	490	**4.2**			1-3	membranes from CHO cells expressing cloned human receptors[66]
4-DAMP	1.02	7.08	**0.56**			1	rat cortex[50]
						2	rat heart[50]
						3	rat submandibular gland[50]
	0.6	3.8	**0.5**	1.17	1.05	1-5	cloned human cells expressed in CHO cells[67]

Supplementary Table 2. Muscarinic receptor homology modeling templates for 3D structure reconstruction. For each of the 5 subtypes, the table shows the template PDB code and the sequence identity (S.I.).

	M_1	M_2	M_3	M_4	M_5
Template	4DAJ	3UON	4U16	3UON	4DAJ
S.I.	77.27%	97.24%	97.10%	83.50%	78.79%

Supplementary Table 3. Mouse muscarinic receptor *in silico* docking. Summary of the residues involved in the most important interactions with each of four antagonists, arranged by bond type.

Antagonist	M_1	M_2	M_3	M_4	M_5	Interaction
4-DAMP	$D^{3.32}$	$D^{3.32}$	$D^{3.32}$	$D^{3.32}$	$D^{3.32}$	salt bridge
	$Y^{3.33}$ $S^{3.36}$ $Y^{6.51}$	$Y^{3.33}$			$Y^{6.51}$	h-bond
	$V^{3.40}$ $W^{4.50}$ $F^{5.40}$ $T^{5.42}$ $F^{5.47}$ $W^{6.48}$	$W^{4.50}$ $F^{5.40}$ $T^{5.43}$ $Y^{6.51}$ $W^{6.48}$	$Y^{3.33}$ $F^{5.40}$ $T^{5.42}$ $V^{6.55}$	$F^{5.40}$ $T^{5.42}$ $W^{7.34}$	$W^{7.36}$ $F^{5.40}$	hydrophobic
			$Y^{6.51}$	F^{185}		pi-stacking
Telenzepine	$D^{3.32}$	$D^{3.32}$	$D^{3.32}$	$D^{3.32}$	$D^{3.32}$	salt bridge
	$Y^{6.51}$ $N^{6.52}$	$Y^{3.33}$	$Y^{6.51}$ $Y^{7.39}$	$Y^{6.51}$ $Y^{7.43}$ $N^{6.52}$	$Y^{3.33}$ $S^{.57}$	h-bond
	$W^{4.50}$ $Y^{7.39}$	F^{101}	F^{224} L^{224} $T^{5.40}$	F^{188} L^{226} $T^{5.40}$	L^{226} $T^{5.40}$ $W^{6.48}$	hydrophobic
	$Y^{3.33}$					pi-stacking
VU 0255035	$D^{3.32}$					salt bridge
	$S^{3.36}$ $Y^{7.39}$		$Y^{7.39}$	$Y^{6.51}$ $Y^{7.43}$		h-bond
	$W^{4.50}$ $Y^{6.48}$ ECL3 $W^{7.34}$	$W^{4.50}$ $T^{5.41}$ ELC3 $Y^{7.39}$ $Y^{7.43}$	$T^{5.40}$ ECL3 $T^{5.43}$ $W^{7.34}$	ECL2 $T^{5.40}$ $T^{5.43}$	$W^{7.34}$ $T^{5.40}$ $T^{5.43}$ ECL2 $W^{4.57}$	hydrophobic
	$W^{7.35}$	$Y^{3.33}$	$W^{6.48}$	$Y^{3.33}$		pi-stacking
J104129 fumarate	$D^{3.32}$		$D^{3.32}$		$D^{3.32}$	salt bridge
	$Y^{3.33}$ $Y^{6.51}$	$Y^{7.38}$	$Y^{3.33}$ $Y^{6.51}$		$Y^{3.33}$ $Y^{6.51}$	h-bond
	$W^{4.50}$ $T^{5.41}$ $Y^{7.39}$ $W^{7.35}$ $W^{6.48}$	$Y^{3.33}$ $T^{5.41}$ $W^{4.57}$ $Y^{7.39}$	$W^{4.50}$ $Y^{5.41}$ $Y^{6.51}$ $Y^{7.39}$	$I^{2.52}$ $L^{3.31}$ $V^{3.34}$ $W^{4.50}$ $W^{4.54}$	$W^{4.50}$ $T^{5.40}$ $T^{5.43}$ $Y^{7.39}$	hydrophobic
			$Y^{7.39}$			pi-stacking

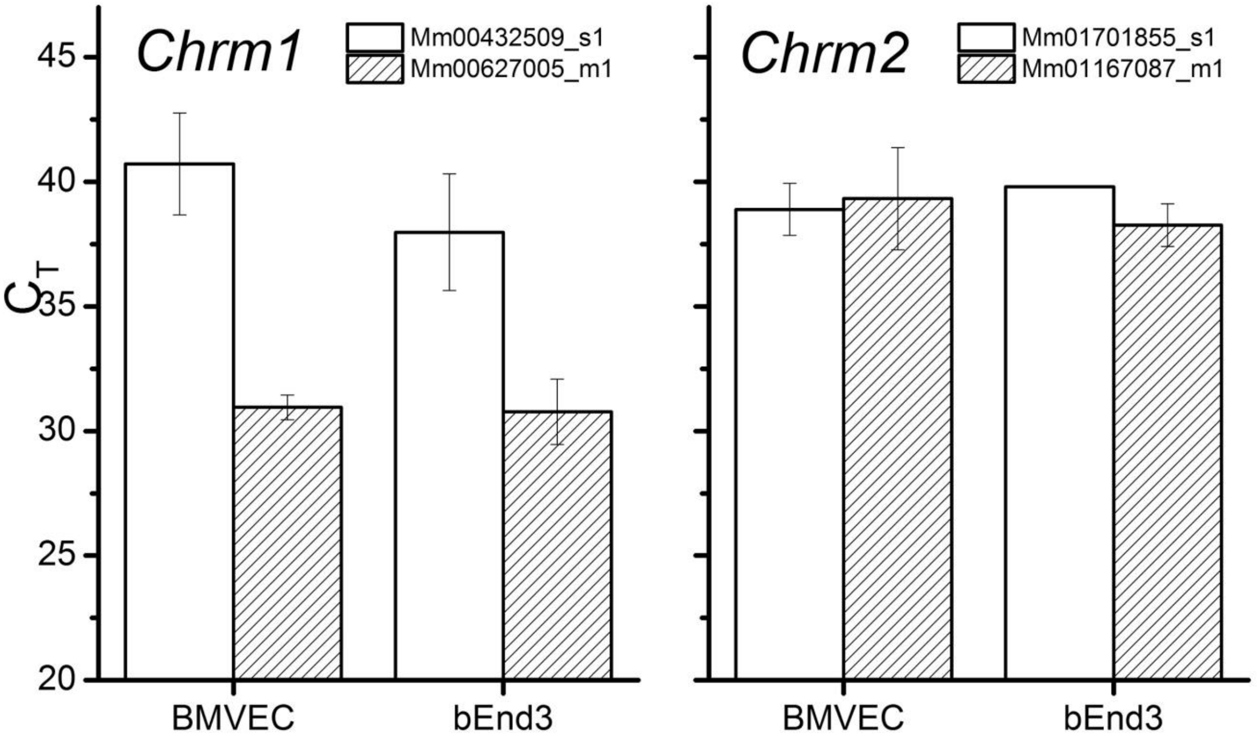

Supplementary Figure 1. Primer screening for optimal quantification of *Chrm1* and *Chrm2* mRNA levels. Comparative threshold cycle (CT) values (mean ± SD, N = 5) are plotted for each tested primer. Even if *Chrm2* primer Mm01701855_s1 (gray shading) reached the threshold at almost the same cycle number as its "white" counterpart, reproducibility was better since the latter resulted in amplification in only 3 out of 9 repetitions. In the end, the "grey" primers were used in generating the data presented in the manuscript (**Figure 1**, see also Methods section).

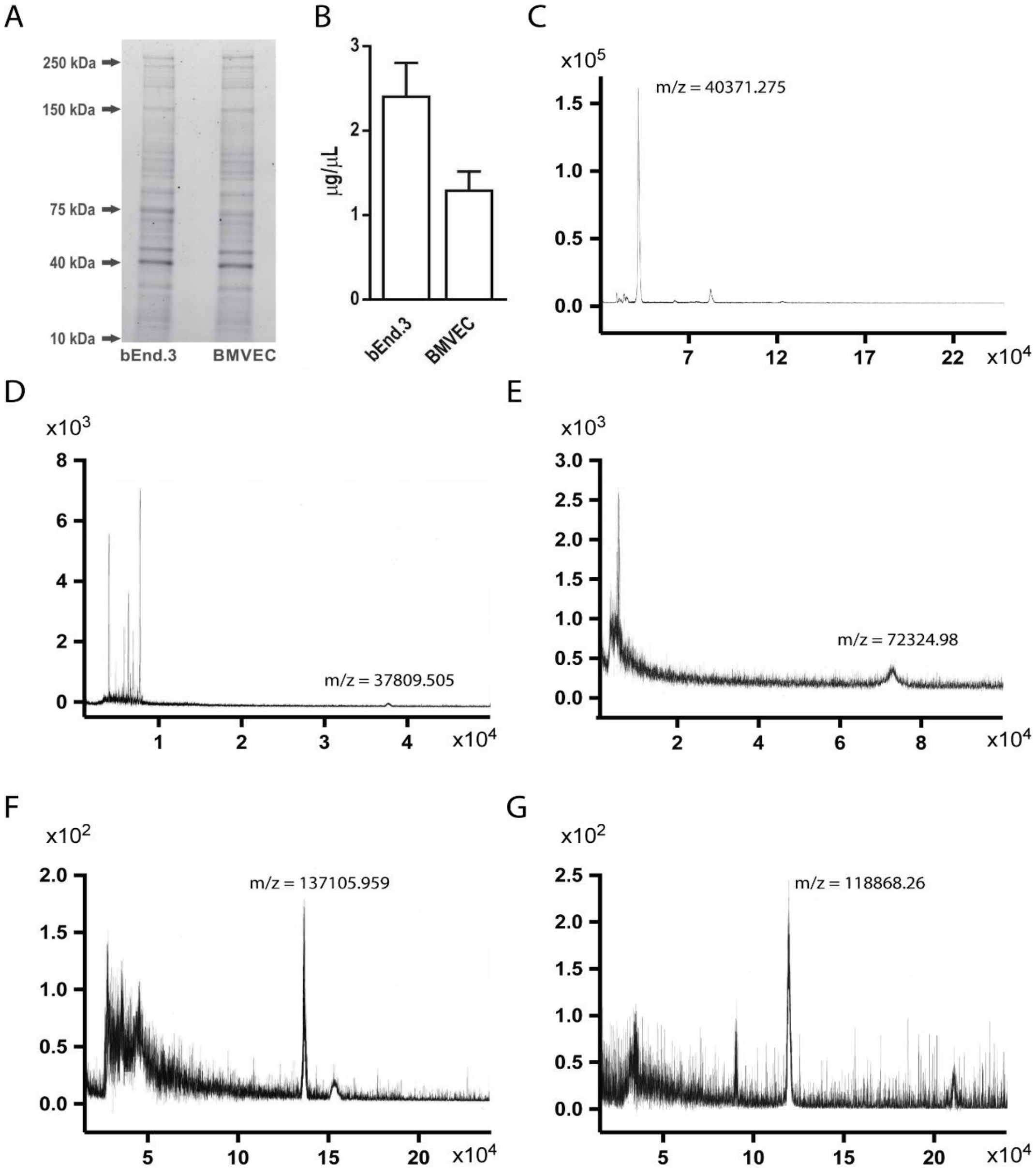

Supplementary Figure 2. Representative examples of stain-free gel and mass spectra. A. Gel electrophoresis of the total proteins extracted from BMVEC and bEnd.3 samples run on 4-15% Criterion™ TGX Stain-Free™ Protein Gels. The visualization of stain-free gels after gel electrophoresis shows well-separated proteins in the range of ~12 to ~200 kDa for both brain endothelial cell extracts. **B.** Total protein content (mean ± SD, N=5) quantified via the Bradford method. Protein levels were significantly higher in bEnd.3 compared to BMVEC samples (unpaired t-test, $t = 8.384$, $df = 18$, $p < .0001$). **C-G.** MALDI-TOF mass spectra of $M_{1\text{-}5}$ receptors in BMVEC samples (identical MS spectra were obtained for bEnd.3 cells). Receptors were identified based on the molecular or fragment peaks in the spectra corresponding to the most abundant, singly charged protein ions. On axis Y - intensity in atomic units, on X - m/z values.

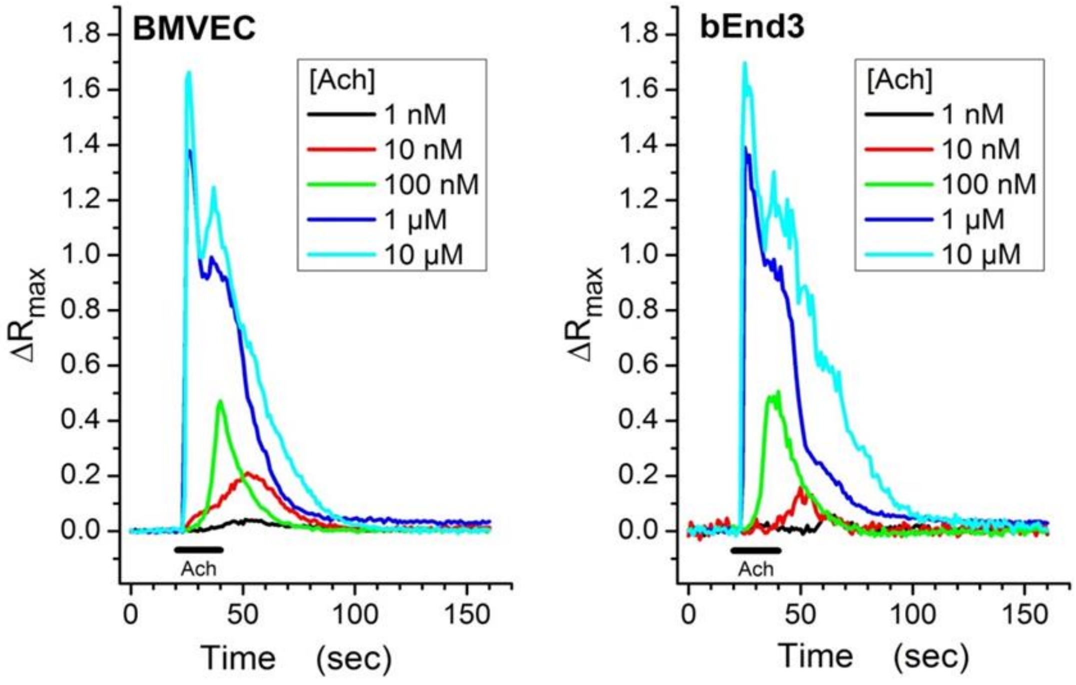

Supplementary Figure 3. Representative traces of mean fluorescence ratio (ΔR) changes induced by acetylcholine in BMVECs and bEnd.3 cells during calcium imaging recordings.

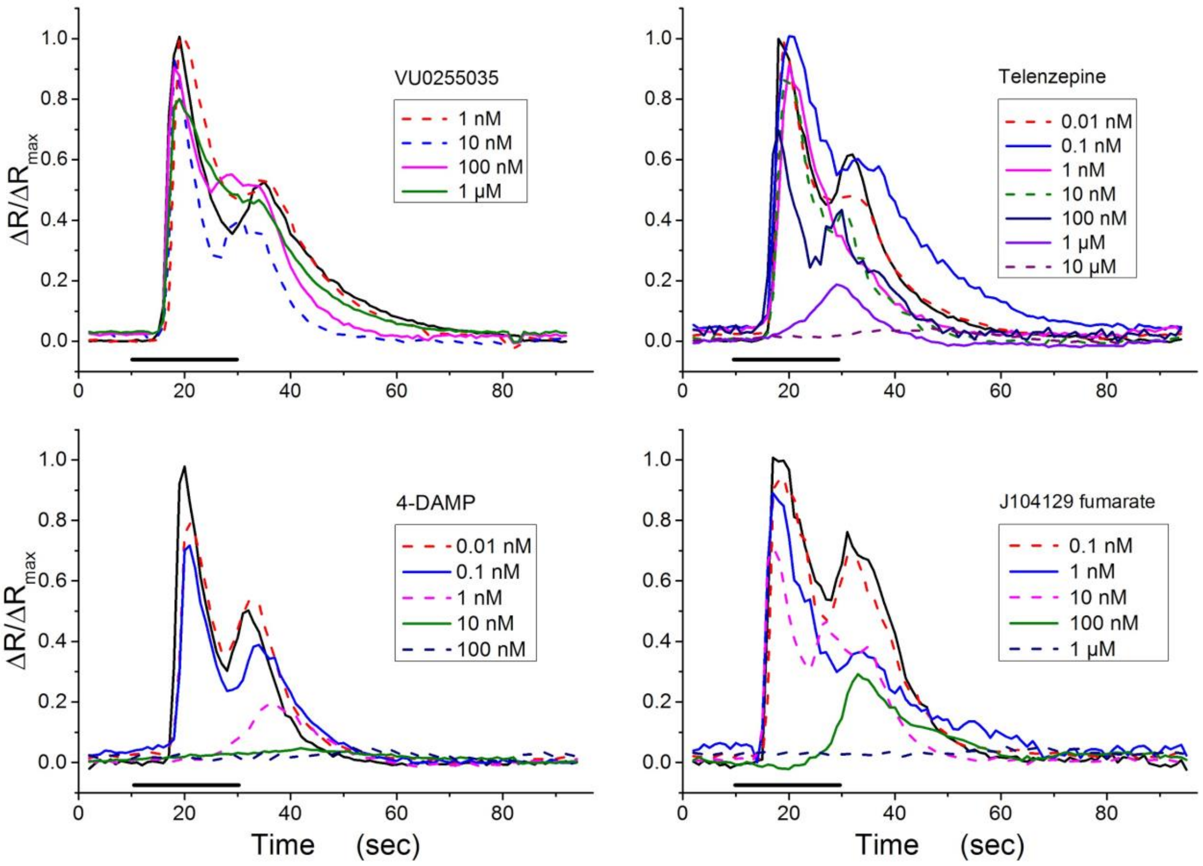

Supplementary Figure 4. The effect of four mAChR antagonists on the ACh (1 μM)-induced calcium signal in bEnd.3 cells. Representative traces of relative fluorescence ratio ($\Delta R/\Delta R_{max}$) changes are plotted. The double-pulse protocol was used, and antagonists were applied 2 min prior to the second ACh pulse (only the second pulse is represented in the graph, and the black bar marks the ACh application period).

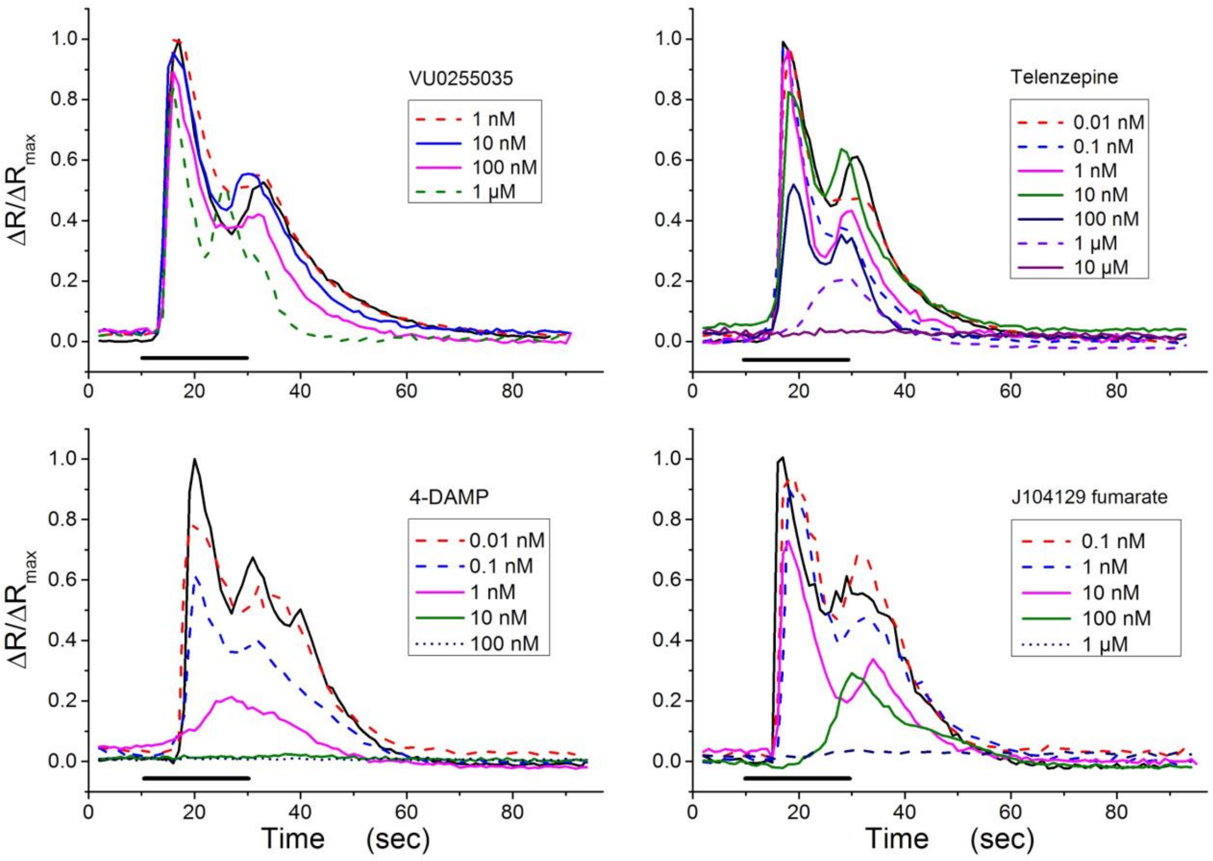

Supplementary Figure 5. The effect of four mAChR antagonists on the ACh (1 μM)-induced calcium signal in BMVEC cells. Representative traces of relative fluorescence ratio ($\Delta R/\Delta R_{max}$) changes are plotted. The double-pulse protocol was used, and antagonists were applied 2 min prior to the second ACh pulse (only the second pulse is represented in the graph, and the black bar marks the ACh application period).

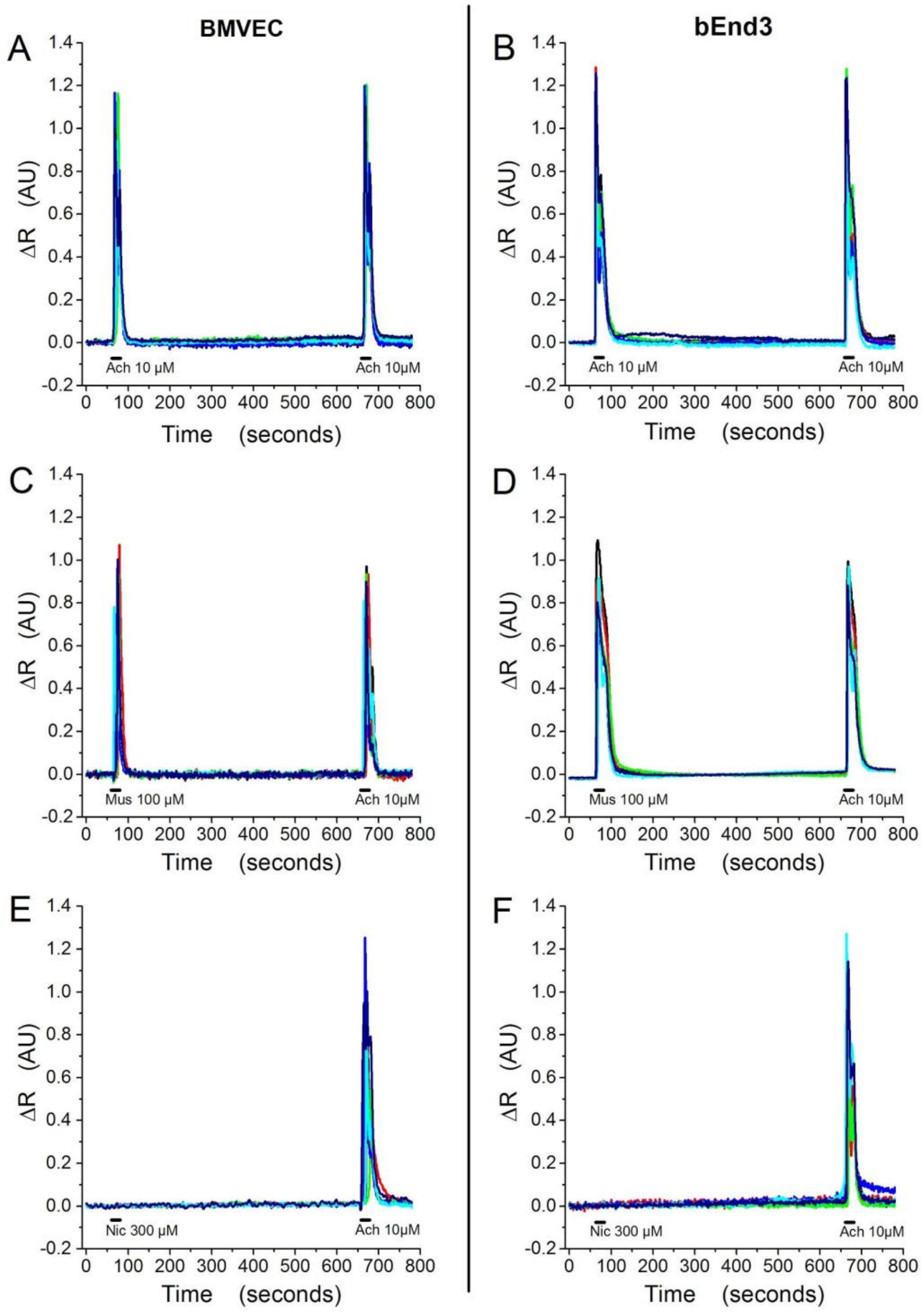

Supplementary Figure 6. Representative traces (one for each of 5 individually recorded cells) of mean fluorescence ratio (ΔR) changes induced by acetylcholine (10 µM) (**A, B**), muscarine (100 µM) (**C, D**), and nicotine (300 µM) (**E, F**) in BMVECs (**A, C, E**) and bEnd.3 cells (**B, D, F**).

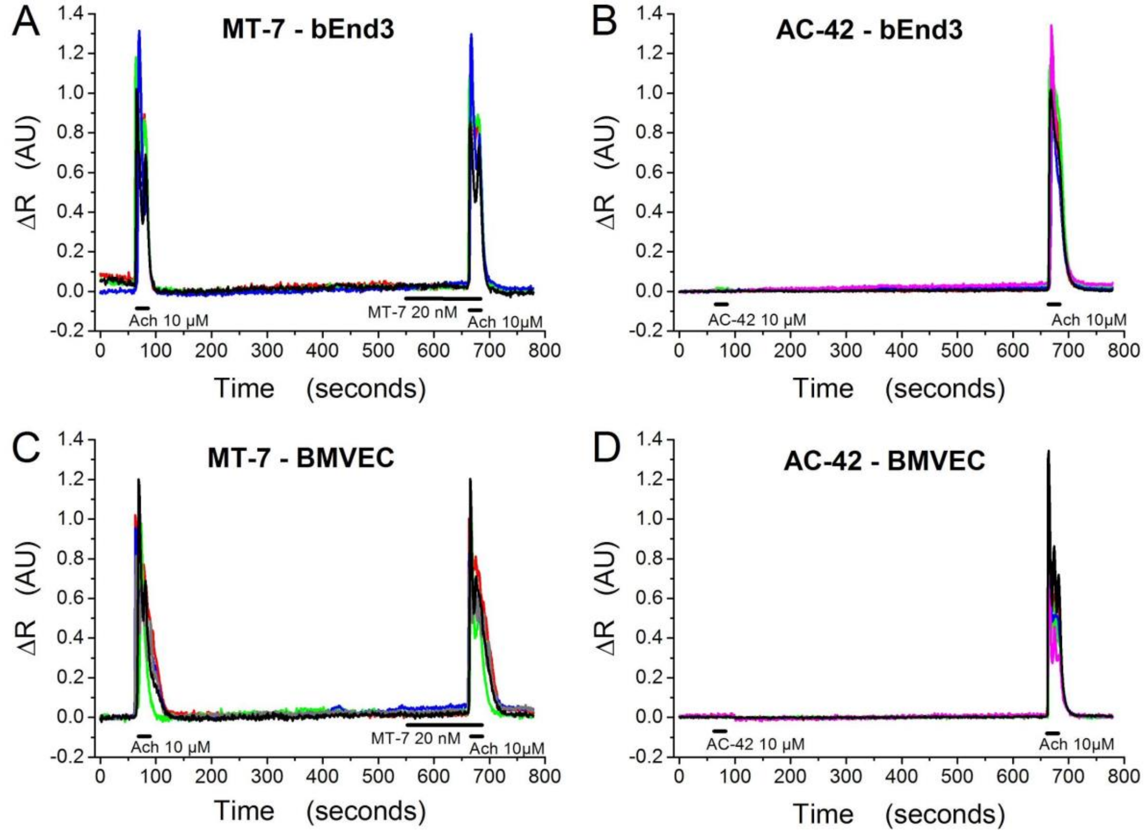

Supplementary Figure 7. Representative traces (5 cells) of mean fluorescence ratio (ΔR) changes induced by MT-7 (20 nM), a potent M_1 antagonist (*A*, *C*), and AC-42 (10 μM), an M_1 specific agonist (B, D) in bEnd.3 cells (*A*, *B*), and BMVECs *(C, D)*.

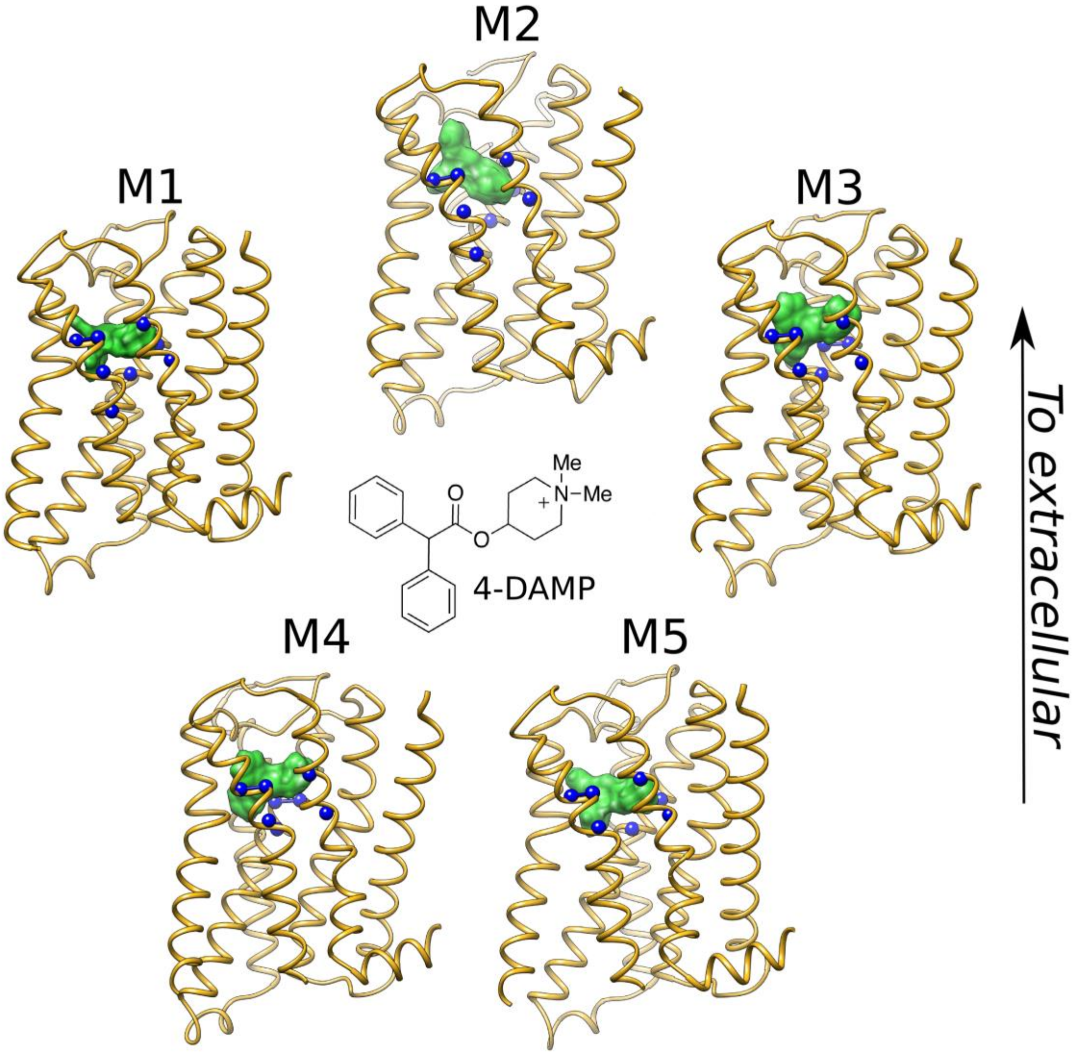

Supplementary Figure 8. 4-DAMP docking in the binding cavity of the M_1-M_5 mouse receptors. C_α of residues in the orthosteric cavity are shown in blue and the antagonist is shown in green within the ribbon representation of the receptor.

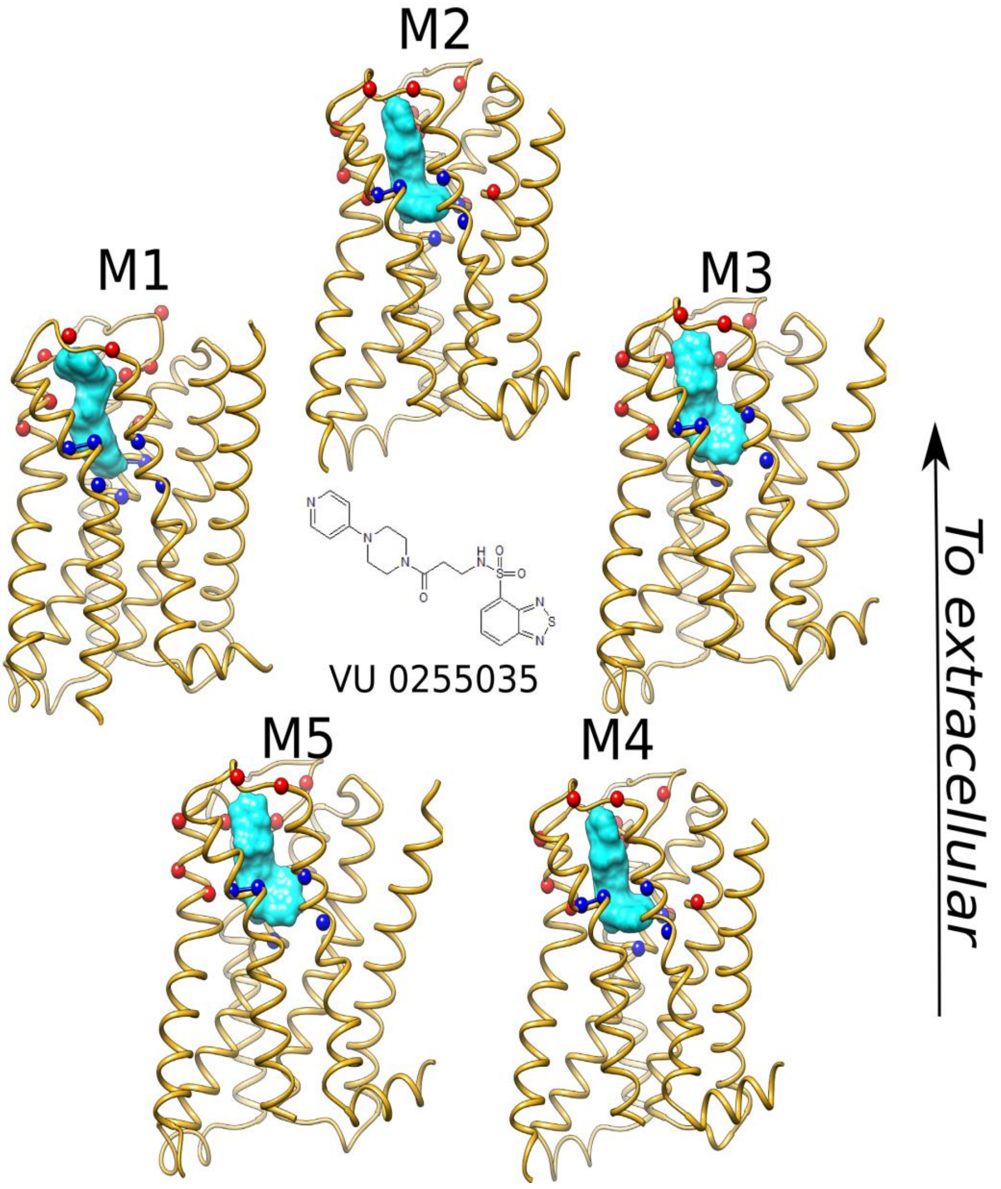

Supplementary Figure 9. VU 0255035 docking in the binding cavity of the M_1-M_5 mouse receptors. C_α of residues in the orthosteric cavity are shown in blue, C_α of residues located in the allosteric cavity are shown in red and the antagonist is shown in cyan within the ribbon representation of the receptor.

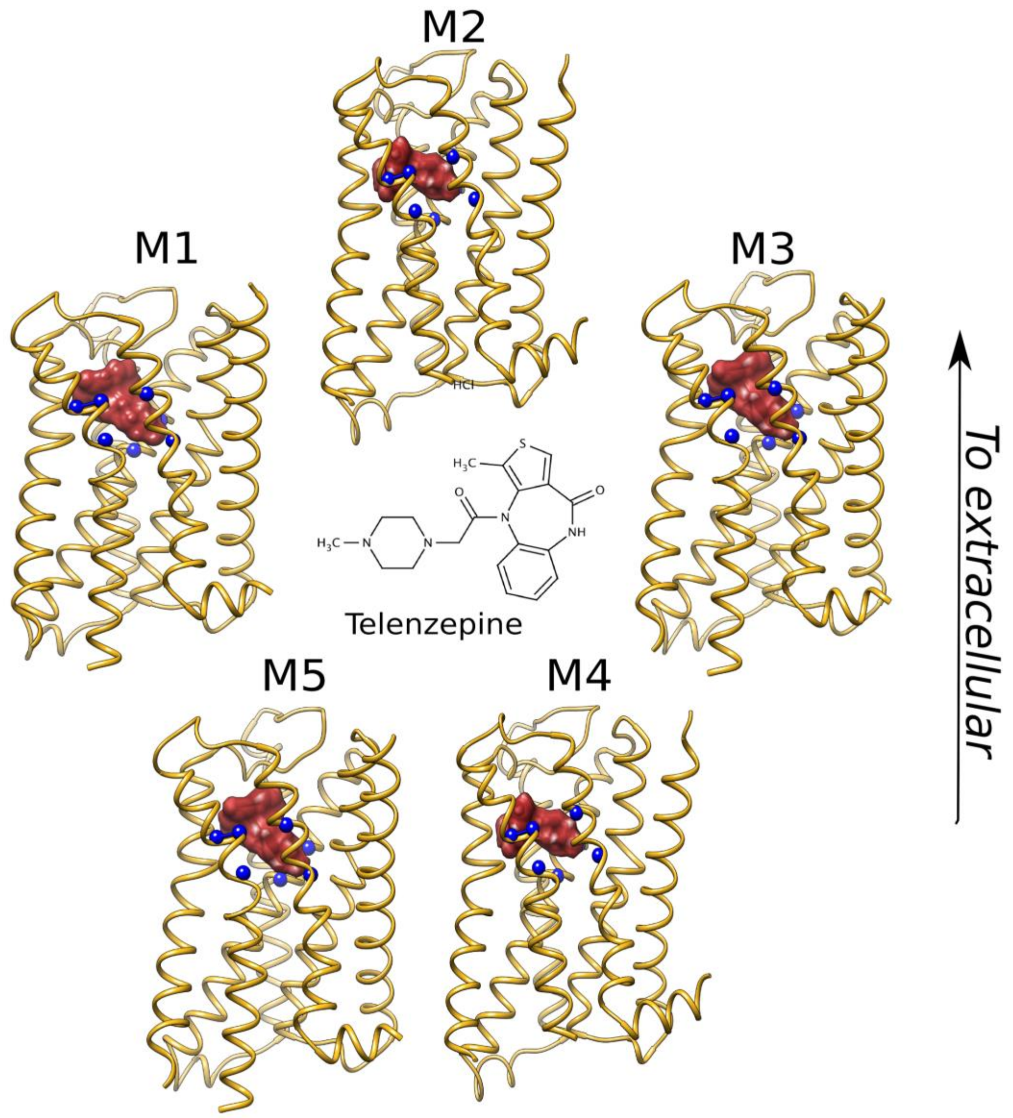

Supplementary Figure 10. Telenzepine docking in the binding cavity of the M_1-M_5 mouse receptors. C_α of residues in the orthosteric cavity are shown in blue and the antagonist is shown in red within the ribbon representation of the receptor.

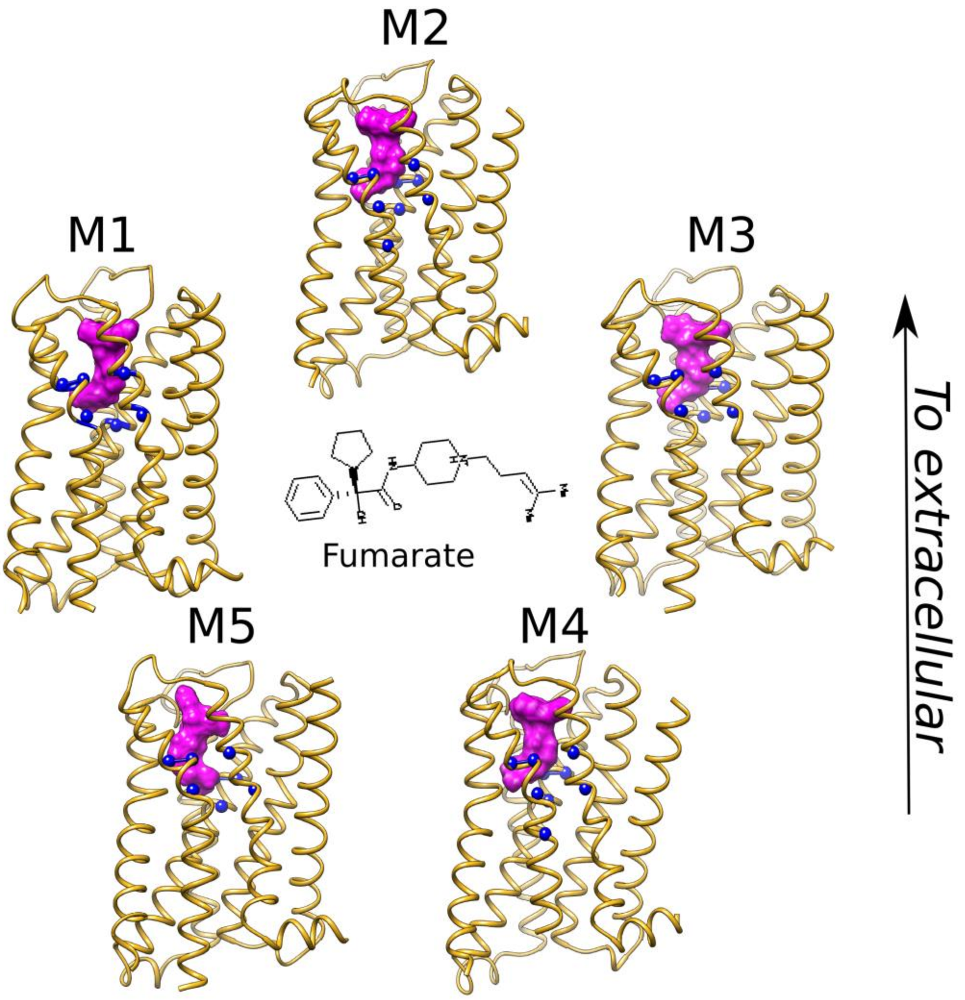

Supplementary Figure 11. J104129 fumarate docking in the binding cavity of the M_1-M_5 mouse receptors. C_α of residues in the orthosteric cavity are shown in blue and the antagonist is shown in magenta within the ribbon representation of the receptor.

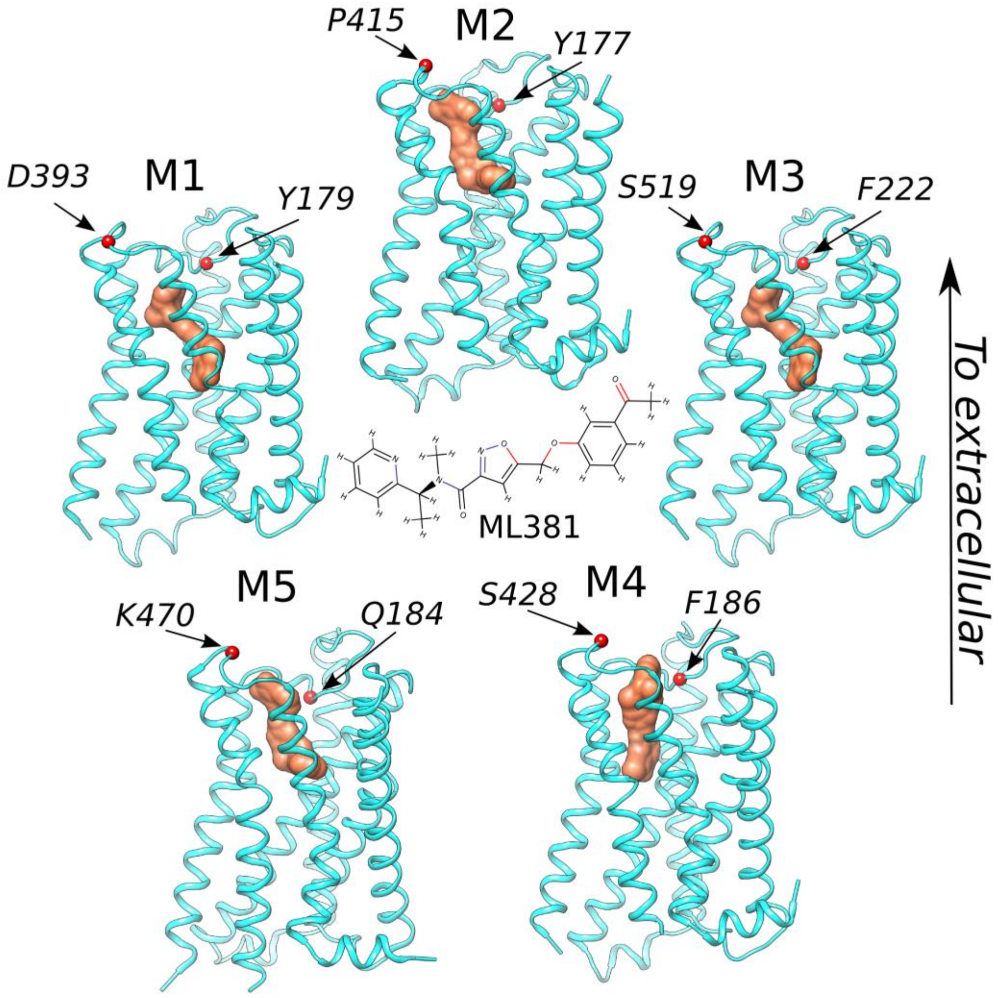

Supplementary Figure 12. VU 0488130 (ML381) docking in the binding cavity of the M_1-M_5 mouse receptors. C_α of residues in the allosteric cavity are shown in red and the antagonist is shown in brown within the ribbon representation of the receptor.
